# Measuring university students’ ability to recognize argument structures and fallacies

**DOI:** 10.3389/fpsyg.2023.1270931

**Published:** 2023-12-06

**Authors:** Yvonne Berkle, Lukas Schmitt, Antonia Tolzin, Andreas Janson, Thiemo Wambsganss, Jan Marco Leimeister, Miriam Leuchter

**Affiliations:** ^1^Institute for Children and Youth Education, Educational Sciences, University of Kaiserslautern-Landau, Landau, Germany; ^2^Information Systems, Research Center for IS Design (ITeG), University of Kassel, Kassel, Germany; ^3^Institute of Information Management (IWI-HSG), University of St. Gallen, St. Gallen, Switzerland; ^4^Institut of Digital Technology Management, Bern University of Applied Sciences, Bern, Switzerland

**Keywords:** measuring argumentation skills, argument structure, fallacies, domain specificity, argument quality

## Abstract

**Theory:**

Argumentation is crucial for all academic disciplines. Nevertheless, a lack of argumentation skills among students is evident. Two core aspects of argumentation are the recognition of argument structures (e.g., backing up claims with premises, according to the Toulmin model) and the recognition of fallacies. As both aspects may be related to content knowledge, students studying different subjects might exhibit different argumentation skills depending on whether the content is drawn from their own or from a foreign subject. Therefore, we developed an instrument to measure the recognition of both argument structures and fallacies among the groups of preservice teachers and business economics students in both their respective domains (pedagogy and economics), and a neutral domain (sustainability). For the recognition of fallacies, we distinguished between congruent and incongruent fallacies. In congruent fallacies, the two aspects of argument quality, i.e., deductive validity and inductive strength, provide converging evidence against high argument quality. In incongruent fallacies, these two aspects diverge. Based on dual process theories, we expected to observe differences in the recognition of congruent and incongruent fallacies.

**Aims:**

We investigated whether these two abilities are domain-specific and whether the recognition of fallacies depends on the congruence of two aspects of argument quality.

**Methods:**

267 preservice teachers and 56 business economics students participated in the study. For the recognition of argument structures, participants assigned the five statements constituting one argument to the corresponding component according to the Toulmin model. For the recognition of fallacies, we created arguments and incorporated a common fallacy into some of them: formal fallacy, overgeneralization, irrelevance, or circularity. Participants rated whether the argument was cogent or not, which was followed by a brief justification.

**Results:**

Domain specificity could not be found for either of both abilities. For the recognition of fallacies, two dimensions were found: a congruent dimension (formal fallacies and overgeneralizations) and an incongruent dimension (irrelevance and circularity).

**Discussion:**

The instrument measures the recognition of both argument structures and fallacies in these two groups across domains. The recognition of fallacies differs depending on whether the deductive validity and the inductive strength of the argument are equally indicative of argument quality or not.

## 1 Introduction

Argumentation is a fundamental aspect of human communication and thought (Mercier and Sperber, 2011). Uses of argumentation include, among others, epistemic caution and the interindividual regulation of information through persuasive communication (Mercier, 2012). By presenting evidence and reasoning, people can make their ideas clear and compelling and can engage in constructive dialog with others or build shared mental models (van den Bossche et al., 2011). However, this communicative function is closely related to an epistemic function (Lumer, 2005). To this end, argumentation represents the rational effort to choose among different options for potential solutions to problems. By presenting evidence and reasoning in support of one of the options and evaluating the arguments of others, people can develop their critical thinking skills and learn to evaluate claims and ideas more effectively (Mercier, 2017).

### 1.1 The structure of arguments

For a long time, the focus of argumentation theory was laid on formal logic and on the development of syllogisms in order to distinguish cogent arguments from non-cogent ones. Accordingly, an argument structurally consists of premises from which a conclusion is logically derived (Copi, 1998). In formal logic, arguments are expressed in a symbolic, formal language. In everyday use, however, arguments are usually expressed in natural language and refer to a particular content and context. For informal arguments, the formal evaluation of arguments is not sufficient (Blair and Johnson, 2000). Therefore, additional criteria have been established for assessing the quality of informal logic in arguments. These criteria consider not only the form, but also the content and context of the argument (Toulmin, 2003).

According to Toulmin (2003), an argument consists of six interrelated components: The *claim* is a statement or an assumption that must be supported by at least two different statements. The *data* serves to explain the validity of the claim, and the *warrant* is an inference rule, that explains the context for why this data supports the claim. A simple argument consists at least of these three components, with the warrant occasionally being omitted when it might be assumed to be common sense or shared knowledge (Bayer, 2007). A simple argument can be extended by adding a *backing*, which additionally supports the validity of the warrant, or a *rebuttal*, which constrains the validity of the claim, thus showing that the boundaries of the argument have been taken into consideration. The sixth component, known as the *qualifier*, does not stand as an independent statement. Instead, it is a word used to signify the level of confidence or certainty associated with the claim, for example, “certainly” or “probably (Toulmin, 2003).

Example:

Claim “There are probably dogs around.”
Data “You can hear it barking and howling.”
Warrant “Dogs are animals that bark and howl.”
Backing “The neighbor has two German shepherd dogs.”
Rebuttal “There could also be wolves.”

In sum, theories on formal and informal logic aim to describe how the premises of an argument rationally support a conclusion or a claim (van Eemeren et al., 2013).

Evaluating an argument requires distinguishing these components as well as some degree of reasoning about the link between the claim and its premises (Bayer, 2007). In particular, recognizing the claim and the warrant are crucial to any understanding or further evaluation of the argument (Britt and Larson, 2003; Richter and Maier, 2018). Nevertheless, students face difficulties with regard to recognizing claims and premises in arguments (Britt et al., 2007, 2014; von der Mühlen et al., 2019). In a study by Britt et al. (2007), university students first rated their level of agreement with arguments and were then asked to immediately recall the claim. Approximately 25% of the participants could not recall the claim correctly. Additionally, university students achieved only 30% accuracy in finding claims in a reading and searching task and neglected warrants (Larson et al., 2004). This finding is in line with the conclusions of Münchow et al. (2020), who found that university students struggled to recognize the components of an argument, especially with regard to identifying claims and warrants. In the context of our study, where our goal is to assess university students’ ability to identify argument structures and fallacies, participants were instructed to identify the *claim* of an argument. Moreover, as students even show difficulties to distinguish claims from premises, we decided not to differentiate between data, warrant, and backing, but refer to them as supporting *premises*. Furthermore, *rebuttal* was differentiated from supporting premises as it constrains the validity of the claim. Hence, the distinction was between claim, support (data, warrant, and backing), and rebuttal. The ability to recognize the argument structure is a prerequisite for evaluating the quality of the argument (Bayer, 2007; Byrnes and Dunbar, 2014; Christodoulou and Diakidoy, 2020). To this end, different types of reasoning have been distinguished (Rips, 2001).

### 1.2 Deductive and inductive reasoning

Arguments can be formal or informal. Formal arguments are expressed in a formal, logical language that represents the logical form of an argument, independent of its content. An informal argument is expressed in natural language with meaningful content and within a meaningful context (Blair and Johnson, 2000).

Informal arguments can be transformed into formal arguments to explicate their logical form (Brun and Hirsch Hadorn, 2009). Informal and formal arguments both consist of a conclusion (which corresponds to the claim in model of Toulmin, 2003) and at least one premise, in which context the premises provide support for the conclusion (Walton, 2005). An argument is cogent when the premises plausibly explain the conclusion, and the conclusion can be derived from the premises using logical reasoning (Walton and Macagno, 2015; Macagno et al., 2018). Each argument should be seen as an external representation of internal reasoning. We categorize different types of reasoning, with our study focusing on deductive and inductive reasoning, which will be explained in the following sections.

#### 1.2.1 Deductive reasoning

In deductive reasoning, cogent arguments follow formal logical rules that are independent of their content, and the truth of the premises guarantees the truth of the conclusion (Bayer, 2007). In non-cogent deductive arguments, the premises cannot guarantee the truth of the conclusion because the whole argument has a logically invalid form (Copi, 1998). For example, the formal argument “If A, then B. A. Therefore, B” is deductively valid. That is, it is not possible for both premises to be true but the conclusion to be false. However, the argument “If A, then B. Not A. Therefore, not B.,” is deductively invalid. The conclusion cannot be deduced from the premises, since there may be other causes of B than A; thus, the argument’s form is invalid. This holds independently of which contents are substituted for A and B (Salmon, 2006). Even if further premises, such as “If A, then B. If X, then B. A. X. Therefore, B”, are added, a deductively valid conclusion cannot become invalid (Copi, 1998). This characteristic is related to the fact that deductive inferences are monotonic, meaning that the addition of new conclusion-contravening information cannot alter a previously derivable conclusion (Kienpointner, 1996). Therefore, a deductive argument is more suitable for the application of prior knowledge than for the development of new knowledge. Since the content of the conclusion is entirely contained within the content of its premises, one can infer that, when the premises are known to be true, the conclusion of a deductively valid argument is known to be true as well. Hence, the conclusion serves as application of prior knowledge to a particular case in order to organize prior knowledge and to explain or predict certain circumstances (Bayer, 2007).

However, in natural communication, arguments are usually informal, and cogent arguments do not guarantee the truth of their conclusions. For instance, considering the degree of certainty of the claim, e.g., expressed by the word “probably,” the argument may still be cogent even if it has a deductively invalid form (“If A, then B. Not A. Therefore, probably not B.”), as discussed in the Bayesian approach (e.g., Hahn et al., 2005). Moreover, the addition of a third premise (“If C, then not B.”) that contradicts the first conclusion may invalidate this first conclusion (“If A, then B. A and C are. Therefore, B.”). Furthermore, the addition of further premises (“If X is C, then X is not B.”) may weaken the argument or make it invalid, if premises are added that contradict a previous premise (“If X is A, then X is B. If X is not A, then X is B. Therefore, X is B”; Copi et al., 2014). Therefore, the mere consideration of the logical form is not sufficient for the evaluation of the quality of an argument. The content and the context also have to be considered, as in inductive reasoning (Hitchcock, 2007).

#### 1.2.2 Inductive reasoning

Inductive arguments follow an informal logic that does consider the content and context of an argument (Johnson, 1999). The conclusion does not necessarily follow from the premises in a formal way, but the premises provide support for the probability of the conclusion being true (Copi et al., 2006, 2014).

Example: “Using cotton shopping bags is not more environmentally friendly than plastic bags, as many people think. Although they decompose faster, they require many more resources to produce.”

The premise in this example, i.e., that the production of the bags must be considered in addition to their disposal, does not guarantee the truth of the conclusion. It does, however, provide a plausible explanation (people who assess the environmental friendliness do not consider the amount of resources needed to produce them) for why many people overestimate the environmental friendliness of cotton bags and thus makes this conclusion more likely. In this case, the argument is cogent, although it is not deductively valid. Hence, the cogency of an inductive argument does not depend exclusively on the logical form of the argument, as in the case of deductive arguments, but also on its content. An informal argument is inductively stronger the more its premises indicate a high probability that the conclusion is true (Backmann, 2019). Unlike deductive arguments, adding more premises can increase or decrease the inductive strength of inductive arguments (Salmon, 2006), meaning that inductive arguments are not monotonic. Since the conclusion goes beyond what has already been stated in the premises, they are also suitable for acquiring new knowledge. Inductive reasoning plays an important role in learning processes because, unlike deductive reasoning, it allows for hypotheses generation (Bayer, 2007). Inductive reasoning involves finding rules from a series of observations that can be used to draw conclusions about a general entity. Where inductive reasoning allows for hypothesis generation, abductive reasoning allows for hypothesis testing as well (Thagard, 2007). However, since abductive reasoning is not of interest to our study, we will not discuss it further.

Although an informal argument does not require deductive validity, an inductively weak argument can still be deductively valid, such as in irrelevant or circular arguments, which will be explained in the following section (Walton, 1992; Hahn et al., 2005). This fact makes it difficult to distinguish high quality arguments from fallacies (Davidson, 2001).

### 1.3 Fallacies

A fallacy is a deductively valid or invalid argument that reflects a conclusion where the premises do not provide the stated (evidential) support for the claim or conclusion (Hamblin, 1970). Fallacies thus impair the quality of an argument (Walton, 2004). There are many possible fallacies; hence, we are not able to present a consistent classification or typology (Hamblin, 1970). However, in the context of our study, we consider four fallacies, which we frequently observed when correcting our students’ essays: formal fallacy, overgeneralization, irrelevance, and circularity.

#### 1.3.1 Formal fallacy

In formal fallacies, the argument does not instantiate a valid argument form, which makes the argument deductively invalid (Brun and Hirsch Hadorn, 2009). This does not mean that the premises or the conclusion are false but rather that they are incorrectly linked (Salmon, 2006). Formal fallacies are not related to inductive reasoning, as content and context are not considered.

Example: “In experiments in which learners were asked to choose among tasks of varying difficulty, individuals with high confidence in success were significantly more likely to choose difficult tasks. From this fact, it can be concluded that learners with low confidence in success would be more likely to choose easier tasks.”

Translated into formal language, this claim reads as follows: “If A (individuals have high confidence in success), then B (they choose difficult tasks). Not A (individuals have low confidence in success). Therefore, not B (they choose easier tasks).” This example illustrates a formal fallacy, known as denying the antecedent. This type of fallacy stands in contrast to the following informal fallacies, which depend on content and context rather than from form (Löffler, 2008).

#### 1.3.2 Overgeneralization

An overgeneralization is a non-cogent argument that is deductively invalid and simultaneously inductively weak, as it does not provide sufficient evidence to support the conclusion (Johnson and Blair, 2006). Instead of sufficient evidence, it is based on anecdotal evidence or a small sample size; as a result, the argument makes a generalization that is not supported by the evidence (Bayer, 2007).

Example: “Students of energy technology who attended an additional seminar on the finite nature of water as a resource and the importance of saving water in everyday life went on to behave more sustainably in other areas of their lives. Thus, including a seminar on some aspect of sustainability in all undergraduate programs would encourage students to become more environmentally conscious in general.”

#### 1.3.3 Irrelevance

An irrelevant argument has premises that are not related to the core issue on which the conclusion focuses (Hurley, 2011). It may rather distract from the core issue, e.g., through personal attacks or by shifting the issue, instead of arguing relevantly (Damer, 2009). Such arguments are inductively weak because they make no substantive contribution to the core issue. These fallacies are difficult to recognize because they often seem to be cogent at first glance, as the argument may be deductively valid (Walton, 2004).

Example: “Due to the increasingly swift digital change, the ability to think logically and in a problem-solving manner is becoming increasingly important. This is already evident in pioneering countries such as England and Australia, where logic and problem solving have been integrated into the curricula from the first grade onward.”

In this example, the premise that other countries have already introduced such subjects into their curricula is not a relevant evidence to support the claim that digital change has heightened the importance of the ability to think logically.

#### 1.3.4 Circularity

Circular arguments are deductively valid but inductively weak because the conclusion is substantiated only by itself (“C. Therefore, C”), albeit expressed in other words (Rips, 2002). A statement that cannot be accepted without further evidence and therefore requires argumentative support cannot simultaneously be accepted as a reason to conclude that the statement is true (Copi, 1998). Circular reasoning is also difficult to recognize as fallacious because the argument initially appears to be cogent due to its deductive validity (Hahn et al., 2005).

Example: “In recent years, controllers in most companies have increasingly acted as advisors to management regarding process flows or as initiators and facilitators of change and learning processes. Therefore, they are increasingly assuming a consulting function as business partners for management in complex business and product-related issues.”

In summary, fallacies affect the quality of an argument in different ways, i.e., formal fallacies through deductive invalidity and informal fallacies through inductive weakness. Nevertheless, when assessing an informally fallacious argument, deductive validity can be considered as well: overgeneralizations are not deductively valid, fallacies of irrelevance may be deductively valid and circular arguments are deductively valid. Thus, to distinguish fallacies from cogent arguments, both deductive validity and inductive strength should be considered.

Research has shown that university students evaluate arguments intuitively based on their prior attitudes and beliefs, thereby neglecting appropriate criteria (Shaw, 1996; Wu and Tsai, 2007; Jonassen and Kim, 2010; von der Mühlen et al., 2016). Accordingly, arguments are more likely to be accepted as cogent if they are consistent with (and thus confirm) one’s prior beliefs, even if the argument is fallacious (Markovits and Nantel, 1989; Klaczynski et al., 1997; Sá et al., 1999; Macpherson and Stanovich, 2007). Such spontaneous evaluations and the subsequent acceptance of fallacious arguments occur even when these prior beliefs are not very strong (Diakidoy et al., 2015, 2017). Von der Mühlen et al. (2016) investigated the strategies used by first-semester psychology students to evaluate arguments and showed that 44% of all judgments were the result of intuitive judgments, while only 12% were due to judgments of relevance and sufficiency.

Dual-process theories serve as a possible explanation for this tendency toward confirmation-biased judgments (Christodoulou and Diakidoy, 2020). Such theories suggest that there is a rapid, heuristic (Type I) process and a slower, strategic (Type II) process for evaluating arguments. Type I is based on the automatic activation of prior knowledge and beliefs in associative memory. The demands on the individual’s cognitive resources thus remain low, which in turn increases the individual’s susceptibility to biased judgments and the acceptance of fallacies due to faulty and incomplete prior knowledge and personal beliefs (Stanovich and West, 2008). A conscious, strategic process is necessary to overcome intuitive, automatic associations. This Type II process requires not only more time but also more cognitive effort (Evans, 2007; Stanovich et al., 2008). Type I is more likely to influence the evaluation of the inductive strength, while Type II is more likely to influence the evaluation of the deductive validity of an argument (Heit and Rotello, 2010).

Based on dual-process theories, the evaluation of an argument’s cogency may differ depending on their congruence with respect to deductive validity and inductive strength (Table 1). We assume that congruence is given when the two aspects of argument quality (*deductive validity* and *inductive strength*) are convergent. In this regard, we distinguish congruent fallacies from incongruent fallacies. Formal fallacies and overgeneralizations are congruent, as they are neither deductively valid nor inductively strong. Thus, both aspects converge. In contrast, fallacies of circularity and fallacies of irrelevance are incongruent, as they are deductively valid but inductively weak. Arguments without fallacies are incongruent as well, since they are deductively invalid but inductively strong. Moreover, as prior knowledge has been shown to be a core element of dual-process theories, domain-specific aspects must be considered.

**Table 1:** Aspects of argument quality.

Fallacy	Premise for the claim: “For students with high expectation in success, motivation is the highest for very difficult tasks.”	Deductive validity	Inductive strength
Formal fallacy	“The motivation for students without high confidence in success must be higher in less difficult tasks.”	−	−
Overgeneralization	“See Paul, who always choose the most difficult tasks, as he knows, that he is able to solve them.”	−	−
Irrelevance	“It is simply more challenging to work on a difficult task if you are highly interested in the topic.”	+	−
Circularity	“If they know to be able to solve the task, they are more likely to rise to the challenge, even if it is a difficult task.”	+	−

### 1.4 Argumentation skills in different domains

Argumentation is applied across domains (Klahr et al., 2011) and is a particularly important skill for all scientific disciplines. Formal logic, a branch of mathematics, has long been the main perspective on cogent argumentation (e.g., Russell and Whitehead, 1910; Scheuer et al., 2010). More recently, however, theories have also focused on practical human argumentation (e.g., Kuhn, 1992; Toulmin, 2003). Although researchers have reached a consensus that argumentation involves both domain-general and domain-specific aspects, it is still unclear which aspects are domain-specific and which are domain-general (Kelly and Takao, 2002; Sampson and Clark, 2008; De La Paz et al., 2012; Barstow et al., 2017; Daxenberger et al., 2018; Klopp and Stark, 2020). Studies investigating the influence of domain-specific knowledge on argumentation skills have reported different results.

In a pre-post study by Zohar and Nemet (2002), students who received instruction on domain-specific content improved their content knowledge but not their argumentation skills. Students who received additional instruction regarding argumentation skills also improved their argumentation skills. Thus, a sole focus on domain-specific knowledge did not improve argumentation skills. This finding is not in line with the results of a qualitative study by Sadler and Zeidler (2005), who showed that participants with high subject-specific knowledge also performed better in argumentation than subjects with low subject-specific knowledge.

A possible explanation for these contrasting findings is provided by the *Threshold Model of Content Knowledge Transfer* (Sadler and Donnelly, 2006). This model suggests that the relationship between domain-specific knowledge and reasoning ability is not linear; rather, content knowledge affects reasoning ability at two different thresholds. The first threshold is passed when so-called “rules of game knowledge” is achieved. This term refers to basic knowledge, such as a basic vocabulary or a general understanding of the most basic concepts. The second threshold is based on differences in how experts, as opposed to novices, process information. This processing includes an understanding of relevant scientific concepts beyond what can be expected of a high school graduate but can be understood by university students studying the respective subject (Sadler and Donnelly, 2006).

The *Threshold Model of Content Knowledge Transfer* posits that the argumentation skills of university students should increase when transitioning from the content of a foreign field of study (no content knowledge) to a common everyday topic (first threshold passed) and finally to their own field of study (second threshold passed). However, with regard to the argumentation skills of university students, the fact that these skills are not as high as expected or required has often been criticized (Klopp and Stark, 2020).

### 1.5 Measuring argumentation skills

Although argumentation is relevant in all subjects of study and a general lack of argumentation skills has been reported, standardized instruments for analyzing and assessing argumentation skills are rare (Rapanta et al., 2013). The most common method used to evaluate arguments involves qualitative analysis or the use of category systems or rating scales to assess students’ argumentative texts (Schwarz, 2003; Jonassen and Kim, 2010; Hefter et al., 2014; Barstow et al., 2017; Ackermann and Kavadarli, 2022). These approaches offer deep insights, but they are also very time-consuming. Standardized test instruments are therefore better suited for large samples.

The *Argument Evaluation Test* (AET, Stanovich and West, 1997) measures the ability to judge the strength of argumentative statements in fictional dialogs on a rating scale. Expert judgments are used as an objective measure of evaluation. Larson et al. (2009) used the Flawed Judgment Test (FJT) to measure the ability to discriminate between structurally acceptable and unacceptable arguments. Both the AET and the FJT refer to general rather than subject-specific topics, such as political or social issues.

With the *Argumentation Competencies Test* (ACT), Klopp and Stark (2020) developed a standardized, subject-specific instrument that focuses on fallacies in the sense of misinterpretations of statistical results. To measure the ability to recognize typical fallacies, Münchow et al. (2019) developed the *Argument Judgment Test* (AJT). This test involves, first, judging the plausibility of given arguments and, second, assigning arguments that are judged to be implausible to common fallacies in a multiple-choice format. The *Argument Structure Test* (AST, Münchow et al., 2020) measures the ability to recognize the structural components of an argument in line with the model of Toulmin (2003). Short texts, each of which consists of one argument containing all five components, are first presented, and then each statement as a segment must be assigned to the corresponding component in a multiple-choice format. Both the AJT and the AST refer to content pertaining to psychological topics.

### 1.6 Rationale of the research and research questions

Our purpose is to measure university students’ ability to recognize argument structures and fallacies. To the best of our knowledge, no standardized instrument has yet been developed to measure the skills required to recognize both, argument structures and fallacies, across different domains. Therefore, we developed the *Argumentation Fallacies and Structures Test* (A-FaST) to measure these two argumentative abilities across three domains (i.e., pedagogy, economics, and sustainability) in the present study.

The model of Toulmin (2003) serves as a framework to investigate university students’ argumentative skills. Based on this framework, we examine whether university students can recognize an arguments’ claim and distinguish it from its premises. Moreover, our goal is to assess students’ ability to recognize fallacies in informal arguments.

More specifically, with our test, we aim to explore whether different fallacies represent two dimensions of argument quality, i.e., deductive validity and inductive strength. Moreover, the distinction between congruent and incongruent fallacies allows for a more specific examination of deductive validity and inductive strength (see dual-process theories, Rips, 2001; Evans, 2007). In congruent fallacies, both aspects of argument quality converge; in incongruent fallacies, they diverge. With our test, we aim to distinguish between the ability to recognize congruent (formal fallacies and overgeneralizations) and incongruent fallacies (irrelevance and circularity).

According to the *Threshold Model of Content Knowledge Transfer* (Sadler and Donnelly, 2006), argumentation abilities depend on the level of prior knowledge (almost no knowledge, basic knowledge, and expert knowledge). We assume university students to have expert knowledge in a domain related to their own field of study, almost no knowledge in a domain related to a foreign field of study and basic knowledge in a neutral, everyday domain. Thus, our purpose is to measure the recognition of argument structures and fallacies among students of different fields of study (preservice teachers and business economics students) in three domains (subject-specific, non-subject-specific, and neutral). Therefore, the A-FaST includes two domains related to the respective fields of study (pedagogy, economics) and one neutral domain (sustainability) for both groups of students.

Furthermore, to examine item difficulty and participant’s latent ability in recognizing argumentation structures and fallacies, we use Item Response Theory (IRT). This allows us to score every participant’s ability based on the applied set of items.

The aim of this study is to validate the A-FaST and to answer the following research questions (RQ):

RQ1a, RQ1b: Is it possible to identify a consistent factor structure of the abilities to recognize (a) argument structures and (b) fallacies in argumentation when examining students from two different fields of study (i.e., preservice teachers and business economics students)?
RQ2a, RQ2b: Are the abilities to recognize both (a) argument structures and (b) fallacies three-dimensional constructs with respect to students’ prior knowledge in the different domains (pedagogy, economics, and sustainability)?
RQ2c: Is the ability to recognize congruent and incongruent fallacies a two-dimensional construct when considering deductive validity and inductive strength?
RQ3a, RQ3b: Do the items related to the assessment of the recognition of (a) argument structures and (b) fallacies in argumentation validly measure students’ abilities when applying Item Response Theory (IRT)?

## 2 Methods

### 2.1 Participants

In total, 437 university students participated in the study. A total of 114 participants were removed from further analyses because of missing values. Out of these, 84 participants left the course early. Besides, 30 participants did not respond to the open-ended questions in the fallacy items. We excluded these participants from the study to rule out motivation biases. Thus, 323 participants were included in the sample (*M* = 23.45; *SD* = 3.12). Of these participants, 267 were preservice teachers (*M* = 23.33; *SD* = 3.04) and 56 were business economics students (*M* = 24.04; *SD* = 3.44). The study was conducted in different courses in the curricula of the two study subjects under investigation. Descriptive statistics of the participants are summarized in Table 2. The courses were mandatory, but all students participated in this research voluntarily and could withdraw from participation until the end of the study. The data were anonymized before analysis, and the conduct of the study was approved by the ethics committee.

**Table 2:** Sociodemographic information.

		Gender			Grade		Semesters	
	n	f	m	d	M	SD	M	SD
Business economics students	56	29	27	0	2.44	0.42	5.80	2.69
Preservice teachers	267	215	46	1	2.55	0.51	5.98	2.60
Total	323	244	73	1	2.53	0.49	5.95	2.61

Grade = Grade on the university entrance qualification according to the German grading system, which ranges from 1 (very good) to 6 (failed); Semesters = Number of subject-specific semesters of study.

### 2.2 Item construction and selection

The A-FaST encompasses two facets of argumentation abilities: (a) recognizing argument structures and (b) recognizing fallacies. For both abilities, we created items ranging across three different domains: pedagogy, economics, and sustainability. Thus, for both groups of students, argumentation skills were assessed in their own field of study (subject-specific, i.e., pedagogy or economics), in a foreign field of study (non-subject-specific, i.e., economics or pedagogy), and in the context of a common everyday domain (neutral, sustainability).

For the *recognition of argument structures*, we developed 15 items (five for each domain) consisting of five statements that represented the components of one extended argument according to Toulmin (2003): claim, data, warrant, backing and rebuttal (*cf.* Supplementary material 1). Arguments tend to have some order, in which the claim usually comes first or last. Our goal was not to investigate whether participants could identify an appropriate order for the components of an argument, but whether they could distinguish between them. Therefore, the five statements of an argument were presented in a randomized order. This allowed us to measure the ability to recognize components of an argument without being biased by the order. To ensure that the tasks were not too difficult for the participants, we distinguished between claim, support (data, warrant, and backing), and rebuttal which students were required to recognize in the context of the argument as a whole. The five components of an argument were thus condensed into three components from the students’ point of view. Each item provided partial credit: Participants received two points if they correctly assigned all five statements to the three components, one point for accurately assigning four out of five statements, and zero points for any other assignments.

For the *recognition of fallacies,* we developed 18 items (six for each domain, one of which was a simple argument and five of which were extended arguments). A single item consisted of a short text representing an argument (*cf.* Supplementary material 2). The simple arguments consisted of one *claim* supported by one *data* and one *warrant*. The extended arguments additionally contained one *backing* and one *rebuttal* (see Toulmin, 2003). For each domain, four of the extended arguments were erroneous, as they included either formal fallacy, overgeneralization, irrelevance, or circularity. Fallacies of irrelevance have been formulated to be deductively valid in order to demonstrate non-congruence between deductive validity and inductive strength. The remaining two items (one simple and one extended) represented arguments based on inductive reasoning that lacked any fallacies (see Table 3). This framework led to the presence of two congruent items (formal fallacies and overgeneralizations) as well as four non-congruent items (simple and extended arguments with no fallacies, irrelevant arguments and circular arguments). For each item, students decided whether the given text represented a cogent argument (single-choice). They were required to justify this answer briefly by completing the following sentence: “This is (not) a cogent argument because…” If the single-choice section of the item was answered incorrectly, zero points were awarded. Participants were awarded one point only if the single-choice part was answered correctly and an adequate justification was provided in the subsequent open response.

**Table 3:** Overview of the items for the recognition of fallacies.

Items	Argument type	Fallacy
FaP1, FaS1, and FaE1	Simple	No fallacy
FaP2, FaS2, and FaE2	Extended	No fallacy
FaP3, FaS3, and FaE3	Extended	Circularity
FaP4, FaS4, and FaE4	Extended	Overgeneralization
FaP5, FaS5, and FaE5	Extended	Formal fallacy
FaP6, FaS6, and FaE6	Extended	Irrelevance

Fa, Fallacy; P, Pedagogy; S, Sustainability; and E, Economics.

Justifications were rated as adequate if they included criteria suggesting a reasonable evaluation of the quality of the argument. It did not matter whether the specific fallacy of the respective item was correctly identified or named. Answers that indicated errors other than the intended fallacy were also accepted as adequate as long as a relation to deductive validity or inductive strength was apparent. Thus, adequate justifications are represented by statements such as the following:

- …the structure is logical, and the claim is justified with scientific evidence.”
- …you cannot infer one result from another.”
- …it is conclusive but repetitive.”

In contrast, answers were rated as inadequate (zero points) if they indicated that no suitable criteria were used to assess the argument quality. These responses mainly included statements indicating a confirmation bias, for example, by presenting a counterargument, personal (dis)agreement, or reasons that were entirely unrelated to an assessment of cogency, such as the following:

- …I do not think it is, but the other way around.”
- …I have read something similar about it.”
- …it is an important issue.”

The interrater reliability for open answers was high with Cohen’s κ = 0.92.

### 2.3 Procedure

The data were collected in curricular online courses from the participants’ respective fields of study. First, the participants answered the 18 items for recognizing fallacies, followed by the 15 items for recognizing argument structures. As the items for recognizing argument structures contain information about the proper form of arguments, we presented them as the second part of the test to avoid influencing responses to the items for recognizing fallacies. In each part, the items for the three different domains (pedagogy, economics, and sustainability) were presented in blocks. To avoid sequential effects, these blocks as well as the items within each block were presented in a randomized order. After completion of the test, an additional questionnaire concerning sociodemographic information was completed.

### 2.4 Statistical analyses

For statistical data analysis, we used the program R version 4.1.0 (R Core Team, 2023) with the support of the R packages “psych” (Revelle, 2022) for item statistics, “lavaan” (Rosseel, 2012) for the assessment of dimensionality and multigroup confirmatory factor analyses (MGCFA) and “difR” (Magis et al., 2010) for the identification of differential item functioning (DIF). DIF analyses are used as a prerequisite for item response theory (IRT) scaling on the one hand and to indicate domain-specific dimensionality on the other hand (Opitz et al., 2022). Furthermore, the package “TAM” (Robitzsch et al., 2022) was used to perform IRT scaling and person-item maps.

## 3 Results

### 3.1 Argument structures

#### 3.1.1 Item characteristics

Latent reliability was high, with ω = 0.83, 95% CI = [0.80; 0.86]. Item difficulty parameters were low, with the highest value being 0.51 (see Table 4). Thus, this part of the test was difficult for the participants to answer. The item discrimination values were in a good range for most of the items. Only two items (StP3 and StE3) fell below the cutoff. StP3 was correlated with only one other item, so we excluded it. StE3 was correlated with several items (see Table 5), and we assumed that the low discrimination was at least partially related to its high difficulty. Despite the very low difficulty value of 0.04, its discrimination value was 0.15. Hence, we assumed that the item would exhibit better item discrimination in a sample with higher capability and decided to keep this item in the test.

**Table 4:** Item statistics for recognizing argument structures.

Item	Mean [0–2]	SD	Item difficulty	Item discrimination	α if deleted
StP1	0.44	0.73	0.22	0.31	0.81
StP2	0.78	0.87	0.39	0.30	0.81
StP3	0.23	0.53	0.12	0.02	0.82
StP4	1.03	0.89	0.51	0.54	0.79
StP5	0.20	0.54	0.10	0.29	0.81
StS1	0.73	0.89	0.37	0.54	0.79
StS2	0.76	0.90	0.38	0.59	0.79
StS3	0.61	0.83	0.30	0.33	0.81
StS4	0.59	0.83	0.29	0.53	0.79
StS5	0.57	0.88	0.28	0.59	0.79
StE1	0.49	0.79	0.24	0.42	0.80
StE2	0.44	0.75	0.22	0.50	0.80
StE3	0.07	0.34	0.04	0.15	0.81
StE4	0.59	0.81	0.29	0.55	0.79
StE5	0.43	0.75	0.21	0.49	0.80

St, Argument structures; P, Pedagogy; S, Sustainability; E, Economics.

**Table 5:** Correlations among the items for recognizing argument structures.

	StP1	StP2	StP3	StP4	StP5	StS1	StS2	StS3	StS4	StS5	StE1	StE2	StE3	StE4
StP2	0.14^*^													
StP3	−0.03	0.00												
StP4	0.24^***^	0.26^***^	0.07											
StP5	0.15^**^	0.01	0.12^*^	0.21^***^										
StS1	0.17^**^	0.18^**^	−0.02	0.39^***^	0.12^*^									
StS2	0.27^***^	0.26^***^	0.01	0.34^***^	0.26^***^	0.45^***^								
StS3	0.12^*^	0.20^***^	0.07	0.22^***^	0.06	0.24^***^	0.19^***^							
StS4	0.21^***^	0.18^**^	−0.02	0.31^***^	0.27^***^	0.34^***^	0.45^***^	0.16^**^						
StS5	0.17^**^	0.26^***^	−0.08	0.38^***^	0.1	0.44^***^	0.45^***^	0.23^***^	0.40^***^					
StE1	0.21^***^	0.17^**^	0.00	0.30^***^	0.18^**^	0.31^***^	0.28^***^	0.13^*^	0.31^***^	0.28^***^				
StE2	0.13^*^	0.14^*^	0.11	0.38^***^	0.23^***^	0.33^***^	0.33^***^	0.15^**^	0.29^***^	0.39^***^	0.18^**^			
StE3	0.06	0.02	−0.03	0.00	0.11	0.10	0.14^*^	0.02	0.14^*^	0.17^**^	0.04	0.04		
StE4	0.22^***^	0.15^**^	−0.01	0.34^***^	0.23^***^	0.33^***^	0.45^***^	0.25^***^	0.35^***^	0.45^***^	0.22^***^	0.37^***^	0.22^***^	
StE5	0.14^*^	0.07	0.03	0.28^***^	0.16^**^	0.35^***^	0.24^***^	0.25^***^	0.35^***^	0.41^***^	0.32^***^	0.41^***^	0.08	0.33^***^

Computed correlation used the Pearson method with listwise deletion. ^*^p ≤ 0.05; ^**^p ≤ 0.01; ^***^p ≤ 0.001; St, Structure; P, Pedagogy; S, Sustainability; E, Economics.

#### 3.1.2 IRT scaling: local independence and measurement invariance

As a prerequisite for the intended IRT scaling, the absence of local independence and DIF was investigated. Local independence was tested using the adjusted form of Q3 of Yen (1984), as specified in the TAM package (Robitzsch et al., 2022). None of the values were higher than the commonly used range, i.e., between −0.20 and 0.20 (Chen and Thissen, 1997), thus supporting the assumption of local independence. Mean aQ3 = 0.00 and item residual correlations ranging from aQ3 = −0.19 to aQ3 = 0.17.

RQ1a: Is it possible to identify a consistent factor structure of the ability to recognize argument structures when examining students from two different fields of study (i.e., preservice teachers and business economics students)?

To examine measurement invariance in both groups of students, we analyzed DIF as well as a series of MGCFA. DIF was examined using the Mantel–Haenszel statistics, which provides robust results with regard to small sample sizes (Herrera and Gómez, 2008). Mantel–Haenszel statistics indicated significant DIF for item StP2 between preservice teachers and business economics students. DIF did not support any claims concerning differences in ability level but did indicate a violation of the measurement invariance. This finding gave rise to the assumption that, with the exception of item StP2, the factor structure was the same for both subgroups. To ensure that the test was fair for both groups of students, this item was excluded from further analyses.

In terms of measurement invariance, we also conducted a series of MGCFA, i.e., a configural model, a weak invariance model, a strong invariance model, and a strict invariance model (Hirschfeld and von Brachel, 2014). The results are shown in Table 6. As the Δχ^2^ test is sensitive to sample size (Tucker and Lewis, 1973), differences between the models were assessed with the cut-offs suggested by Chen (2007), who proposed ΔCFI < 0.005 and ΔRMSEA > 0.01 to indicate invariance. Furthermore, items were parceled to account for the small number of observations (e.g., Matsunaga, 2008; Levacher et al., 2021). We built five parcels, with the first parcel consisting of the three pedagogy items, the second parcel consisting of the first three sustainability items, the third parcel consisting of the remaining two sustainability items, the fourth parcel consisting of the first three economics items, and the fifth parcel consisting of the remaining two economics items. The results indicated strict measurement invariance between the two groups of students. However, these results are not quite robust due to the small sample size. Therefore, we refer to the results of the Mantel–Haenszel-statistic to assess measurement invariance. The absence of DIF did not suggest item-specific differences between the groups.

**Table 6:** Tests of measurement invariance for argument structures items.

Model	χ^2^	*df*	*p*(χ^2^)	CFI	ΔCFI	RMSEA	ΔRMSEA	Δχ^2^	Δ*df*	*p*(Δχ^2^)
Configural	14.606	10	0.147	0.992	-	0.053	-	-	-	-
Weak	21.035	14	0.101	0.987	−0.005	0.056	0.003	6.429	4	0.169
Strong	21.644	18	0.248	0.993	0.006	0.035	−0.021	0.608	4	0.962
Strict	24.029	19	0.195	0.991	−0.002	0.040	0.005	3.386	1	0.123

#### 3.1.3 Dimensionality

RQ2a: Is the ability to recognize argument structures a three-dimensional construct with respect to students’ prior knowledge in the different domains (pedagogy, economics, and sustainability)?

We investigated dimensionality in further detail using confirmatory factor analysis (CFA). We assumed the ability to recognize argument structures to be multidimensional in terms of the three different domains (pedagogy, economics, and sustainability). This three-factorial solution exhibited a moderate fit. The chi square test (χ^2^ = 76.061, *df* = 62, *p* = 0.108) as well as the SRMR (0.071) indicated a good fit; however, the CFI (0.93) and RMSEA (0.078, *p* = 0.129) were above the cutoffs proposed by Hu and Bentler (1999). For the one-dimensional solution, similar fit indices could be observed (χ^2^ = 79.149, *df* = 65, *p* = 0.112, CFI = 0.93, RMSEA = 0.077, *p* = 0.128, SRMR = 0.072). The chi-squared difference test did not indicate significant differences between the two solutions (Δχ^2^(3) = 3.16, *p* = 0.367). Therefore, we chose the less restrictive, unidimensional model for IRT scaling.

#### 3.1.4 Partial Credit Model

RQ3a: Do the items related to the assessment of the recognition of argument structures in argumentation validly measure students’ abilities when applying Item Response Theory (IRT)?

We applied a unidimensional Partial Credit Model (PCM) to the data, which indicated a good fit (RMSEA = 0.036, *p* ≤ 0.05, SRMR = 0.071). The EAP reliability of 0.78 was acceptable. Discrepancies in the fit of items to the model were indicated using mean-square residual summary statistics. The outfit is an unweighted measure and corresponds to the average of the standardized residual variance across items and persons. The infit equals the individual variance-weighted residuals. Most studies have used values ranging between 0.70 and 1.30 to indicate that an item measures the latent variable (Smith et al., 2008). The infit and outfit indices were within this range for all items (see Table 7). Only for item StS3 did the outfit slightly exceed the threshold, measuring 1.35. However, this item exhibited a good infit; thus, we assumed a sufficient fit for this item.

**Table 7:** Item difficulties and fit statistics as estimated by the PCM.

Item	Difficulty	SE	Infit	Outfit
StP1 (t1)	1.51	0.14	1.18	1.31
StP1 (t2)	0.81	0.18	1.06	1.18
StP4 (t1)	0.30	0.13	1.02	1.00
StP4 (t2)	−0.38	0.13	0.93	0.91
StP5 (t1)	2.93	0.18	1.09	1.06
StP5 (t2)	0.67	0.24	1.03	0.72
StS1 (t1)	1.32	0.13	0.99	1.03
StS1 (t2)	−0.33	0.14	0.92	0.85
StS2 (t1)	1.25	0.13	0.93	0.93
StS2 (t2)	−0.36	0.14	0.92	0.86
StS3 (t1)	1.32	0.13	1.25	1.35
StS3 (t2)	0.17	0.15	1.12	1.30
StS4 (t1)	1.52	0.14	0.98	1.02
StS4 (t2)	0.04	0.15	0.92	0.83
StS5 (t1)	2.70	0.14	0.91	0.84
StS5 (t2)	−1.17	0.15	0.87	0.78
StE1 (t1)	1.85	0.14	1.08	1.09
StE1 (t2)	0.12	0.16	1.05	0.95
StE2 (t1)	1.74	0.14	0.99	0.95
StE2 (t2)	0.49	0.17	0.97	0.72
StE3 (t1)	3.74	0.26	1.07	1.11
StE3 (t2)	1.28	0.40	1.08	1.01
StE4 (t1)	1.27	0.13	0.95	0.92
StE4 (t2)	0.35	0.16	0.89	0.71
StE5 (t1)	2.02	0.14	0.92	0.82
StE5 (t2)	0.22	0.17	1.01	0.81

t1 = threshold 1 (from 0 to 1 point), t2 = threshold 2 (from 1 to 2 points).

**Figure 1:**
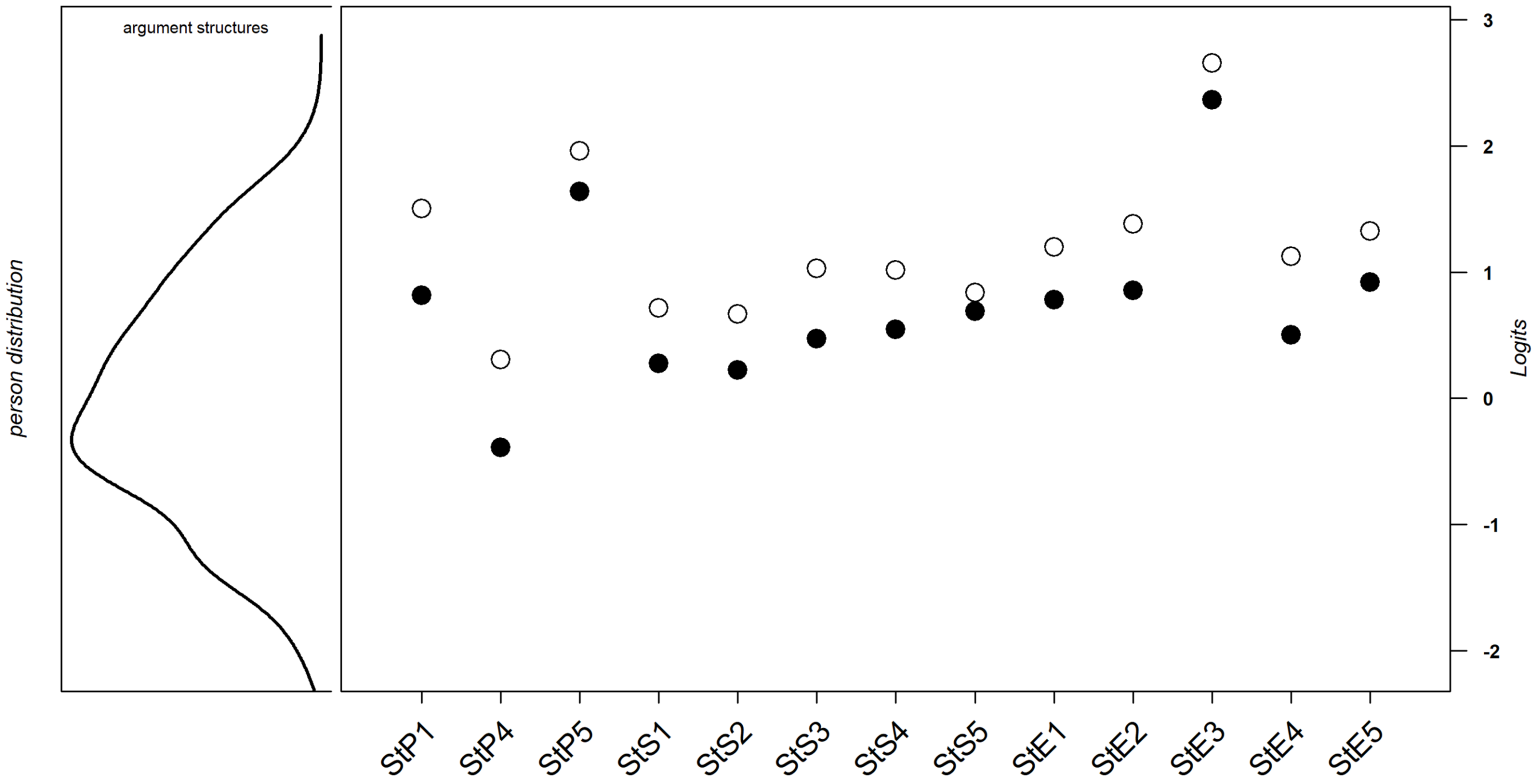
Person-item map for argumentation structure items. St, Structure; P, Pedagogy; S, Sustainability; E, Economics; ●, First threshold; and ⚪, Second threshold.

As the person-item map (Figure 1) shows, the items focused on the medium-to-high ability range of the sample. The lower ability range was not covered by any item. Person abilities were not normally distributed, with more participants in the lower than in the upper ability range.

### 3.2 Fallacies

#### 3.2.1 Item characteristics

Latent reliability was acceptable, with ω = 0.72, 95% CI = [0.68; 0.76]. Item difficulty parameters were low, with the highest value being 0.50 (see Table 8). Thus, this part of the test was difficult for the participants to answer. Item discrimination was also low for some items. We assumed that the high difficulty was at least partly responsible for the observed low discriminatory power and that it might increase in a sample with more expertise. Item FaP4 exhibited the lowest discrimination value of 0.03. Nevertheless, we decided to keep this item. First, the discriminatory power of this item increased to 0.18 when considering only congruent items. Second, it was correlated only with other congruent items and not with incongruent items (see Table 9) and thus seemed to be useful for further evaluation of the dimensionality of this ability.

**Table 8:** Item statistics for recognizing argumentation fallacies.

Item	Mean [0–1]	SD	Item difficulty	Item discrimination	α if deleted
FaP1	0.50	0.50	0.50	0.47	0.68
FaP2	0.42	0.49	0.42	0.37	0.69
FaP3	0.26	0.44	0.26	0.30	0.69
FaP4	0.13	0.33	0.13	0.03	0.72
FaP5	0.11	0.32	0.11	0.11	0.71
FaP6	0.24	0.42	0.24	0.31	0.69
FaS1	0.30	0.46	0.30	0.30	0.69
FaS2	0.44	0.50	0.44	0.43	0.68
FaS3	0.19	0.39	0.19	0.19	0.70
FaS4	0.26	0.44	0.26	0.25	0.70
FaS5	0.39	0.49	0.39	0.26	0.70
FaS6	0.20	0.40	0.20	0.35	0.69
FaE1	0.12	0.33	0.12	0.20	0.70
FaE2	0.40	0.49	0.40	0.21	0.70
FaE3	0.42	0.49	0.42	0.44	0.68
FaE4	0.18	0.38	0.18	0.26	0.70
FaE5	0.29	0.45	0.29	0.32	0.69
FaE6	0.30	0.46	0.30	0.27	0.70

Fa, Fallacy; P, Pedagogy; S, Sustainability; E, Economics; 1&2 = no fallacy, 3 = circularity, 4 = overgeneralization, 5 = formal fallacy, and 6 = irrelevance.

**Table 9:** Correlation matrix for items for recognizing fallacies.

	FaP1	FaP2	FaP3	FaP4	FaP5	FaP6	FaS1	FaS2	FaS3	FaS4	FaS5	FaS6	FaE1	FaE2	FaE3	FaE4	FE5
FaP2	0.31^***^																
FaP3	0.17^**^	0.22^***^															
FaP4	0.01	0.05	−0.02														
FaP5	0.08	0.10	0.06	0.04													
FaP6	0.16^**^	0.06	0.07	−0.06	0.01												
FaS1	0.16^**^	0.20^***^	0.17^**^	0.04	−0.08	0.17^**^											
FaS2	0.20^***^	0.30^***^	0.18^***^	−0.04	0.07	0.17^**^	0.19^***^										
FaS3	0.32^***^	0.02	0.14^*^	−0.09	0.01	0.12^*^	0.15^**^	0.12^*^									
FaS4	0.15^**^	0.07	0.01	0.05	−0.01	0.18^**^	0.00	0.18^***^	0.09								
FaS5	0.13^*^	0.14^*^	0.08	0.19^***^	0.10	0.14^*^	0.00	0.11^*^	0.08	0.18^**^							
FaS6	0.15^**^	0.11^*^	0.13^*^	0.04	0.10	0.22^***^	0.12^*^	0.22^***^	0.08	0.16^**^	0.08						
FaE1	0.20^***^	0.18^***^	0.06	0.00	−0.01	0.15^**^	0.17^**^	0.21^***^	0.11^*^	−0.07	−0.05	0.08					
FaE2	0.28^***^	0.16^**^	0.13^*^	−0.10	−0.05	0.11^*^	0.21^***^	0.23^***^	0.17^**^	0.01	0.06	0.07	−0.01				
FaE3	0.13^*^	0.15^**^	0.22^***^	−0.02	0.04	0.27^***^	0.18^**^	0.22^***^	0.09	0.15^**^	0.17^**^	0.22^***^	0.17^**^	0.15^**^			
FaE4	0.20^***^	0.10	0.11^*^	0.07	0.15^**^	0.07	0.11	0.13^*^	0.09	0.15^**^	0.06	0.16^**^	0.03	0.00	0.12^*^		
FaE5	0.22^***^	0.18^**^	0.12^*^	0.13^*^	0.19^***^	0.16^**^	0.14^*^	0.17^**^	0.06	0.18^**^	0.19^***^	0.20^***^	0.14^*^	−0.12^*^	0.15^**^	0.17^**^	
FaE6	0.22^***^	0.12^*^	0.20^***^	−0.09	0.04	0.11^*^	0.06	0.14^*^	−0.04	0.13^*^	0.09	0.20^***^	0.04	0.13^*^	0.31^***^	0.15^**^	0.07

Computed correlation used the Pearson method with listwise deletion. ^*^p ≤ 0.05; ^**^p ≤ 0.01; ^***^p ≤ 0.001; Fa, Fallacy; P, Pedagogy; S, Sustainability; E, Economics; 1&2 = no fallacy, 3 = circularity, 4 = overgeneralization, 5 = formal fallacy, and 6 = irrelevance.

#### 3.2.2 IRT scaling: local independence and measurement invariance

The assumption of local independence was supported with mean aQ3 = 0.00 and item residual correlations ranging from aQ3 = −0.17 to aQ3 = 0.14.

RQ1b: Is it possible to identify a consistent factor structure of the ability to recognize fallacies in argumentation when examining students from two different fields of study (i.e., preservice teachers and business economics students)?

Mantel–Haenszel statistics indicated significant DIF for the two groups of students (preservice teachers and business economics students) only for one item, i.e., FaE2. This finding gave rise to the assumption that, with the exception of item FaE2, the factor structure was the same for both subgroups. To ensure that the test was fair for both groups, this item was excluded from further analyses.

In terms of measurement invariance, we also conducted a series of MGCFA similar to the argument structures section above. However, our item-based model did not converge, probably due to the small sample size (Yap et al., 2014). Therefore, item parcels were built as well. We built six parcels, with two parcels for each domain, one consisting of the respective four incongruent items of the domain and one consisting of the respective two congruent items of the domain. The results as presented in Table 10 indicated a weak measurement invariance between the two groups of students. However, due to the small sample size, we again refer to the Mantel–Haenszel-statistic to assess measurement invariance. The absence of DIF (with one exception) advocates against item-specific differences between the groups.

**Table 10:** Tests of measurement invariance for fallacy items.

Model	χ^2^	*df*	*p*(χ^2^)	CFI	ΔCFI	RMSEA	ΔRMSEA	Δχ^2^	Δ*df*	*p*(Δχ^2^)
Configural	29.810	12	0.003	0.928	-	0.096	-	-	-	-
Weak	33.299	16	0.007	0.930	0.002	0.082	−0.014	3.489	4	0.480
Strong	40.212	20	0.005	0.918	−0.012	0.079	−0.003	6.913	4	0.141
Strict	63.383	22	≤0.001	0.832	−0.086	0.108	0.029	23.171	2	≤0.001

#### 3.2.3 Dimensionality

To address the dimensionality of the ability to recognize fallacies, we assumed this ability to be multidimensional in terms of the three different domains (pedagogy, economics, and sustainability) or the two different types of argument quality (congruent and incongruent).

RQ2b: Is the ability to recognize fallacies a three-dimensional construct with respect to students’ prior knowledge in the different domains (pedagogy, economics and sustainability)?

The three-factorial model under consideration did not exhibit a good fit according to a CFA (χ^2^ = 383.32, *df* = 119, *p* ≤ 0.001, CFI = 0.39, RMSEA = 0.08, *p* ≤ 0.001, SRMR = 0.118), as none of the fit indices indicated a good fit to the data. Hence, this result did not suggest that a three factorial model featuring the domains as factors was relevant.

RQ2c: Is the ability to recognize congruent and incongruent fallacies a two-dimensional construct when considering deductive validity and inductive strength?

For the assumed two-factorial model with regard to argument quality, a bifactor model including the two factors of congruent and incongruent arguments as well as the general factor of argument quality exhibited a good fit (χ^2^ = 124.28, *df* = 101, *p* = 0.058, CFI = 0.95, RMSEA = 0.03, *p* = 1.0, SRMR = 0.04). The one-factorial solution showed a moderate fit (χ^2^ = 187.29, *df* = 119, *p* ≤ 0.001, CFI = 0.84, RMSEA = 0.04, *p* ≤ 0.001, SRMR = 0.05). However, the two-factorial solution focusing on argument quality fitted the data significantly better (Δχ^2^(18) = 63.02, *p* ≤ 0.001).

#### 3.2.4 1PL testlet model

RQ3b: Do the items related to the assessment of the recognition of fallacies in argumentation validly measure students’ abilities when applying Item Response Theory (IRT)?

We applied a 1PL testlet model with the two factors of congruent (formal fallacy and overgeneralization) and incongruent arguments (no fallacy, irrelevance, and circularity) and one general factor (argument quality), which exhibited a good fit (RMSEA = 0.04, SRMR = 0.05). The overall reliability was high, with ω*_t_* = 0.86. All item difficulties were higher than zero (see Table 11), indicating that they were more likely to be solved by a person with a high ability score. Items with difficulties lower than zero were also more likely to be solved by a person with a low ability score. No obvious differences in difficulty between the two dimensions were observed.

**Table 11:** Item and item fit statistics as estimated using the 1PL testlet model.

Item	Item difficulty	SE	Infit	Outfit
FaP1	0.02	0.13	0.94	0.91
FaP2	0.43	0.13	1.02	0.97
FaP3	1.31	0.14	1.02	0.94
FaP4	2.29	0.18	1.06	1.17
FaP5	2.45	0.19	1.03	1.05
FaP6	1.49	0.14	1.01	0.99
FaS1	1.11	0.13	1.04	1.04
FaS2	0.35	0.13	0.97	0.92
FaS3	1.82	0.15	1.09	1.31
FaS4	1.26	0.14	1.00	0.96
FaS5	0.54	0.13	0.99	0.97
FaS6	1.75	0.15	0.98	0.86
FaE1	2.43	0.18	1.01	0.92
FaE3	0.45	0.13	0.94	0.89
FaE4	1.85	0.16	0.98	0.96
FaE5	1.11	0.13	0.95	0.91
FaE6	1.07	0.13	1.08	1.07

Fa, Fallacy; P, Pedagogy; S, Sustainability; E, Economics; 1&2 = no fallacy, 3 = circularity, 4 = overgeneralization, 5 = formal fallacy, and 6 = irrelevance.

Discrepancies in the fit of items to the model were indicated by the mean-square residual summary statistics. All items exhibited good infit and outfit. Only for item FaS3 did the outfit slightly exceed the threshold with a value of 1.31. However, this item exhibited a good infit; thus, it can also be viewed as exhibiting a sufficient fit (see Table 11).

**Figure 2:**
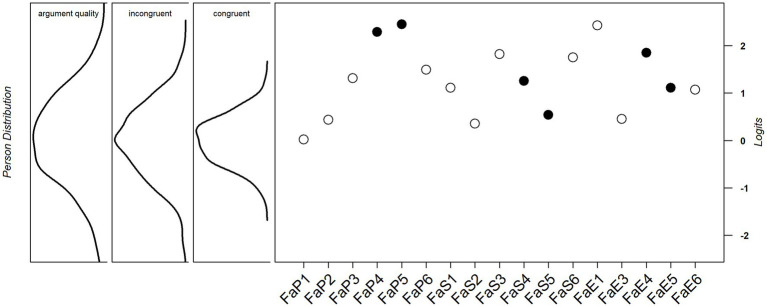
Person-item map for argumentation fallacy items. Fa, Fallacy; P, Pedagogy; S, Sustainability; E, Economics; 1&2 = no fallacy, 3 = circularity, 4 = overgeneralization, 5 = formal fallacy, 6 = irrelevance, ⚪ incongruent, and ● congruent.

Figure 2 shows a person-item map that displays the person distribution on the latent construct visually on the left (general factor: argument quality, dimension 1: incongruent, and dimension 2: congruent) and the location of the item difficulties on the right. The items cover the person abilities in the upper half of the ability distribution well, but no items in the lower ability range of the sample are represented. Items FaP4 and FaP5 are outside the ability range on the dimension 2: congruent.

## 4 Discussion

Our aim was to measure the ability to recognize argument structures and the ability to recognize fallacies among students of different fields of study (preservice teachers and business economics students) in three domains (subject-specific, non-subject-specific, and neutral). For this purpose, we developed the Argumentation Fallacies and Structures Test (A-FaST) across the three domains of pedagogy, economics and sustainability. Pedagogy items are subject-specific for preservice teachers and non-subject specific for business economics students. Conversely, economics items are subject-specific for business economics students and non-subject-specific for preservice teachers. Sustainability items represent a neutral domain for both groups of students.

In RQ1a and RQ1b, we investigated whether it is possible to identify a consistent factor structure of the abilities to recognize (a) argument structures and (b) fallacies in argumentation when examining students from two different fields of study (i.e., preservice teachers and business economics students). With regard to both abilities, DIF was found for only one item each (FaE2 and StP2). We therefore assumed that the A-FaST measures domain-general abilities to recognize structures and fallacies. In the following, we thus excluded these items to ensure that the test was fair for both groups of students. For the remaining items of the A-FaST, the observed factor structures for both abilities apply equally to both groups of students.

In RQ2a and RQ2b, we analyzed whether students’ abilities to recognize (a) argument structures and (b) fallacies are three-dimensional with respect to the different domains (pedagogy, sustainability, and economics). This assumption was based on the *Threshold Model of Content Knowledge Transfer* (Sadler and Donnelly, 2006). The model assumes that the relation between content knowledge and argumentation abilities is not linear. Instead, according to the model, content knowledge affects argumentation abilities at two different thresholds, i.e., between almost no knowledge and basic knowledge, and between basic knowledge and expert knowledge. In the A-FaST, expert knowledge is represented by the respective subject-specific domain, almost no knowledge is represented by the respective non-subject-specific domain, and basic knowledge is represented by the neutral, everyday domain. Accordingly, we expected that preservice teachers and business economics students would differ in terms of their abilities to recognize both argument structures and fallacies in the represented domains according to their study subject but not with regard to the neutral subject. However, for both abilities, this expected domain specificity could not be confirmed by DIF or CFA.

Concerning the three-dimensionality of both abilities, DIF was found only for one item in each case (FaE2 and StP2). We assume that DIF may arise from different levels of prior subject-specific knowledge in both groups of students (Opitz et al., 2022). However, we cannot infer from this result that the A-FaST measures domain-specific abilities, since it pertains to only one item for each ability, each of which is represented in one domain.

Moreover, we conducted CFA to investigate the three-dimensionality (domain specificity) in further detail. Regarding the ability to recognize argument structures, the three-dimensional solution indicated an ambiguous fit. Chi-squared statistics as well as SRMR indicated good model fit, while CFI and RMSEA did not. The one-dimensional solution led to very similar values, and the two models did not differ significantly in terms of fit. Therefore, we adopted the less restrictive, one-dimensional model for this ability. With respect to the ability to recognize fallacies, the CFA for the three-dimensional solution indicated unsatisfactory values for all fit indices. These results of the CFA were in line with the results of the DIF analyses. Thus, our findings fail to supply evidence for adopting a three-dimensional and thus domain specific approach to either ability.

Concerning the ability to recognize fallacies, we posited two-dimensionality with respect to the congruence of the two aspects of argument quality, i.e., deductive validity and inductive strength (RQ2c). Formal fallacies and overgeneralizations are congruent, as they are neither deductively valid nor inductively strong. In contrast, fallacies of circularity and fallacies of irrelevance are incongruent, as they are deductively valid but inductively weak. Arguments without fallacies are incongruent as well, since they are deductively invalid but inductively strong.

Our assumption of such two-dimensionality was based on *dual-process theories* (e.g., Rips, 2001; Evans, 2007). These theories assume the evaluation of an argument’s quality to be affected by two distinct processes, which are often referred to as Type I and Type II processes. Type I is a heuristic process based on an automatic activation of associated prior knowledge and beliefs that occurs unconsciously. In contrast, Type II is an analytical process that involves strategic considerations, is slower and requires more cognitive effort. When evaluating an argument, these two processes may lead to consistent or conflicting results with regard to assessments of argument quality. In cases of conflicting results, a decision must be made in favor of one type when evaluating the quality of an argument. It has been widely assumed that Type I is more likely to influence the assessment of inductive strength, while Type II is more likely to influence the assessment of deductive validity (Heit and Rotello, 2010; Stephens et al., 2018). We therefore assume that judging the quality of congruent arguments differs from judging that of incongruent arguments, which should be crucial with regard to the ability to recognize fallacies. This assumption was supported by the results of our CFA. All fit indices for the two-factor solution (congruent argument quality and incongruent argument quality) with a general factor (argument quality) indicated a good model fit to the data. Therefore, we assume that the ability to recognize fallacies is affected by whether the deductive validity and the inductive strength consistently agree or contradict with argument quality.

In RQ3a and RQ3b, we asked whether all items measure the abilities used to recognize (a) argument structures and (b) fallacies equally well, when applying IRT. Concerning the ability to recognize argument structures, a PCM fit the data well. One item was excluded due to poor discrimination power, and one item was excluded as a result of DIF. For the remaining 13 items, the applied PCM showed that they all measured this ability equally well. All difficulties as estimated by the PCM were in the medium to high ability range of the sample. Furthermore, more subjects were in the lower ability range than in the upper range, confirming the assumption of a deficit in terms of this ability.

For the ability to recognize fallacies, the 1PL-testlet model fit the data well. One item was excluded as a result of DIF. Further analysis showed that the remaining 17 items measure this ability equally well. Item difficulties as estimated using the 1PL-testlet model as well as across the two subsets congruent and incongruent) were all in a medium to high range. The variance in person abilities were lower for the congruent dimension than for the incongruent dimension, as shown by the person-item map (Figure 2). The ability distribution for congruent items was clustered more heavily in the mid-range, while the ability distribution for incongruent items also covered higher and lower abilities. The lower ability range of the general factor argument quality was not covered by any item, thus confirming the assumption of a general deficit with regard to this ability.

### 4.1 Limitations

The sample as a whole performed poorly in both parts of the test, i.e., concerning the ability to recognize argument structures and the ability to recognize fallacies. We aimed to make the test less challenging by focusing on the recognition of the claim, premises, and rebuttal within an argument structure, without differentiating between data, warrant, and backing. The difficulty of these items could be further reduced for the benefit of low-achieving students by using only simple arguments (claim, data, and warrant) and omitting support and rebuttal. Furthermore, the results do not provide information regarding which components were more or less difficult to recognize, which might also be investigated in future studies. Concerning fallacy items, we chose the partially open response format, as this approach offers deeper insights into the criteria that students use to evaluate an argument. Nevertheless, to reduce the difficulty of employing this approach in future research on low-performing samples, the open response part might be replaced by a multiple-choice format.

This poor performance of the sample could be a reason why we did not find domain-specific differences. The students probably had not acquired sufficient domain-specific knowledge in their particular domains to cross the required thresholds according to the *Threshold Model of Content Knowledge Transfer* (Sadler and Donnelly, 2006). To test this assumption in a subsequent study, an additional corresponding knowledge test should be conducted. The wording of the arguments may be another reason that we did not find domain-specificity in either ability. Arguments with content that is not too complex can be understood and evaluated by experts as well as novices (Richter and Maier, 2018). In contrast to the non-DIF items, for both DIF items, the meanings of the key subject-specific, technical terms included in the items (Content Management for the business item (FaE2) and Scaffolding for the pedagogy item (StP2)) are not clarified by the context of the entire argument (*cf.* Supplementary material 2). We assume that without prior knowledge of these terms, it would hardly be possible to follow the argumentation. Thus, the absence of DIF in the remaining items might have been due to the context of the arguments, which sufficiently explained the technical terms used in the arguments.

### 4.2 Conclusion

In summary, the A-FaST was very difficult for participants of the present sample to complete. However, this difficulty might offer the opportunity to track learning gains after an intervention. Moreover, the instrument we developed is less time-consuming than the qualitative assessment of students’ essays and can be conducted in larger groups. Furthermore, it facilitates the comparison of argumentation skills across different domains. Thus, from a measurement perspective, the results of the IRT models revealed the structure and usefulness of the A-FaST for assessing the abilities to recognize both argument structures and fallacies.

## Data availability statement

The raw data supporting the conclusions of this article will be made available by the authors, without undue reservation.

## Ethics statement

The research project has been approved by the Ethical Committee of the Faculty of Educational Sciences at the University of Koblenz-Landau. The studies were conducted in accordance with the local legislation and institutional requirements. The participants provided their written informed consent to participate in this study.

## Author contributions

YB: Conceptualization, Data curation, Formal analysis, Investigation, Methodology, Writing – original draft, Writing – review & editing. LS: Formal analysis, Methodology, Writing – review & editing, Data curation. AT: Conceptualization, Writing – review & editing, Methodology. AJ: Conceptualization, Investigation, Writing – review & editing, Data curation, Methodology. TW: Conceptualization, Investigation, Writing – review & editing, Methodology. JL: Conceptualization, Writing – review & editing, Investigation, Resources. ML: Conceptualization, Funding acquisition, Project administration, Resources, Supervision, Writing – original draft, Writing – review & editing, Investigation, Methodology.
