# Supplementary material for: Measuring university students’ ability to recognize argument structures and fallacies
Source: Front Psychol. 2023 Dec 6;14:1270931. doi: 10.3389/fpsyg.2023.1270931 (PMC10730665; doi:10.3389/fpsyg.2023.1270931)
Supplement: Supplementary file 1 [file Table_1.pdf]

Measuring university students' ability to recognize argument structures and fallacies

Yvonne Berkle, Lukas Schmitt, Antonia Tolzin, Andreas Janson, Thiemo Wambsganss, Jan Marco Leimeister, and Miriam Leuchter

Supplementary Materials

Supplementary Material 1. Items for the recognition of argument structures

Item	Component	German (original)	English (translated)
StP1	Claim	Vor dem Hintergrund eines sozial-konstruktivistischen Lernverständnisses haben verbale Unterstützungsmaßnahmen eine hohe Bedeutung für den Wissensaufbau.	In the context of a social-constructivist understanding of learning, verbal support has a high significance for knowledge acquisition.
	Ground	Die Unterrichtsforschung versteht verbale Unterstützungsmaßnahmen als zentrale Möglichkeit der kognitiven Aktivierung, mit der die aktive und konstruktive Auseinandersetzung mit einem Lerngegenstand gefördert werden soll.	research on classroom practice views verbal support measures as a key feature of cognitive activation, which is intended to promote active and constructive engagement with a learning subject. .
	Warrant	Kognitiv aktivierende verbale Unterstützungsmaßnahmen, die in der frühpädagogischen Forschung im Kontext von Sustained Shared Thinking thematisiert werden, beeinflussen die Qualität von frühpädagogischen Einrichtungen maßgeblich.	Cognitive activating verbal support, in early education research in the context of Sustained Shared Thinking, significantly influences the quality of early education settings.

	Backing	Das Stellen allgemeiner Fragen, z.B. ob bestimmte Dinge bereits bekannt sind, kann zur Aktivierung des Vorwissens von Schülern führen.	Asking general questions, such as whether certain topics are already known, can lead to bridge the gap to students' prior knowledge.
	Rebuttal	Mangelndes fachliches und fachdidaktisches Wissen der Lehrpersonen kann beim Einsatz von verbalen Unterstützungsmaßnahmen zu fachlichen Fehlern führen, die u. U. das Lernen der Kinder behindern.	A lack of teachers’ content-specific and pedagogical content knowledge can lead to content-related errors when using verbal support measures, which may impede children's learning.
StP2	Claim	Für Lehrkräfte ist es wichtig, über grundlegende Kompetenzen des Scaffolding zu verfügen sowie diese im Unterricht adaptiv einzusetzen.	It is important for teachers to have basic scaffolding skills and to use them adaptively during teaching.
	Ground	Das Modell des Scaffolding wurde vielfach empirisch untersucht, dabei hat es sich als zentraler Aspekt der Unterrichtsqualität erwiesen.	The model of scaffolding has been widely studied in empirical research and has shown to be a central aspect of instructional quality.
	Warrant	Im Modell des Scaffolding wird die Transfer of Responsability von Lehrkraft auf Lernenden durch kontinuierliche Kreisläufe von Scaffolding und Diagnose erreicht.	In the model of scaffolding, transfer of responsibility is achieved through continuous scaffolding and diagnosing.
	Backing	Insbesondere verbales Scaffolding hat sich als lernwirksam erwiesen.	Verbal scaffolding, in particular, has shown to be effective to foster (student’s) learning.
	Rebuttal	Scaffolding ist unwirksam, wenn es nicht ausreichend strukturiert ist.	Scaffolding is ineffective if it is not adequately structured.
StP3	Claim	Bereits in der Grundschule können Kinder durch ihr implizites Verständnis für experimentelle Untersuchungen die Notwendigkeit eines kontrollierten Tests zur Hypothesenprüfung erkennen.	As early as elementary school, children can recognize the need for controlling variables in hypothesis testing by building on their implicit understanding of experimental investigations.

	Ground	Untersuchungen zum Einsatz der Variablenkontrollstrategie zeigen, dass Kinder bereits ab der dritten Klasse in der Lage sind, bei Wahlaufgaben einen kontrastiven Test produzieren. Sie wählten Antwortalternativen, die die fokale Variable isolieren und manipulieren und konnten diese Wahl auch mehrheitlich korrekt begründen.	Research on the use of the variable control strategy shows that children in grade 3 are able to design a contrastive test on choice tasks. They have shown to chose response alternatives that isolate and manipulate the variable of interest and were also able to correctly explain the majority of their choices.
	Warrant	Um in multivariaten Umgebungen valide Schlussfolgerungen über Ursache-Wirkungs-Beziehungen zwischen Variablen treffen zu können, ist der Einsatz der Variablenkontrollstrategie nötig. Dabei wird die fokale Variable manipuliert und gleichzeitig alle anderen Variablen konstant gehalten.	To draw valid inferences on cause-effect relationships between variables in multivariate settings, it is necessary to use the variable control strategy. This involves manipulating the variable of interest while holding all other variables constant.
	Backing	Der spontane Einsatz der Variablenkontrollstrategie, der auf ein explizites Verständnis für die experimentelle Überprüfung von Hypothesen schließen lässt, wurde in Untersuchungen erst bei Kindern ab der fünften Klasse beobachtet.	The spontaneous use of the variable control strategy, has been observed in studies only in children in fifth grade and above, which suggests an explicit understanding of experimental hypothesis testing.
	Rebuttal	In der Anwendung der Variablenkontrollstrategie zeigen sich von der Grundschule bis ins Erwachsenenalter deutliche, stabile individuelle Differenzen, die nicht auf eine unterschiedliche Beschulung zurückzuführen sind.	In the use of the variable control strategy, individual differences emerge from elementary school into adulthood that cannot be attributed to differences in schooling.
StP4	Claim	Formatives Assessment ist eines der wirksamsten Instrumente zur Optimierung schulischen Lernens.	Formative assessment is one of the most effective tools for optimizing learning in school.
	Ground	Bereits in den 80er Jahren kamen Bildungsforscher unter Berücksichtigung zahlreicher Forschungsergebnisse zu dem Schluss, dass die mit formativem Assessment zusammenhängenden positiven Effekte auf die Leistung von	Still in the 1980s, educational researchers have concluded that the positive effects of formative assessment on student achievement ranged from d=0.40 to d=0.70.

		Schülerinnen und Schülern zwischen d=0.40 und d=0.70 liegen.	
	Warrant	Durch formatives Assessment wird kontinuierliche Rückmeldung über die Diskrepanz zwischen Lernzielen und individuellem, aktuellem Lernstand gegeben, die den Lernenden dabei unterstützt, den weiteren Lernprozess zu gestalten.	Formative assessment provides continuous feedback on the discrepancy between learning goals and one' current level of proficiency, which helps learners to adapt further learning.
	Backing	Eine aktuelle Übersichtsarbeit, die sich auf die Wirksamkeit von speziell onlinebasiertem formativem Assessment konzentriert, kommt zu dem Schluss, dass dieses eine positive Wirkung auf die Schülerleistung und die Selbstregulation hat.	A recent review focusing on the effectiveness of specifically online-based formative assessment has concluded that it has a positive impact on student performance and self-regulation.
	Rebuttal	Moderatoranalysen zufolge ist die Wirksamkeit von formativem Assessment stark abhängig vom Schulfach, der Schülergruppe sowie der konkreten Umsetzung der Leistungsbeurteilung.	Moderator analyses have shown that the effectiveness of formative assessment strongly depends on the school subject, the student group, and the applied assessment of student performance.
StP5	Claim	Die Förderung der Prozessqualität ist in höchstem Maße herausfordernd.	The promotion of process quality is highly challenging.
	Ground	Studien aus dem Bereich der Elementarstufe zeigen, dass die Prozessqualität in deutschen Kindergärten in Bezug auf den spezifischen Teilaspekt der kognitiv anregenden Interaktionen eher gering ist.	Studies in elementary education haven show that the process quality in German kindergartens is rather low regarding the promotion of cognitive activation.
	Warrant	In diesem Zusammenhang steht zunehmend auch die Rolle der Interaktion zwischen pädagogischen Fachkräften und Kind im Blick der Forschung.	In this context, research has focused more on the interaction between preschool teachers and children.

	Backing	Die Herausforderung zeigt sich in verschiedenen Kompetenzbereichen, wie z. B. in Sprachfördereinheiten und naturwissenschaftlichen Lerngelegenheiten.	The challenge is evident in various content areas, such as in language development and science learning.
	Rebuttal	Es gibt Modelle, nach denen die Prozessqualität gefördert werden kann.	There are models which assume that process quality can be promoted.
StS1	Claim	Es ist nachhaltiger, regional aber konventionell produziertes Obst und Gemüse zu kaufen, als Bio-Produkte, die aus weit entfernten Gegenden kommen.	It is more sustainable to buy regionally but conventionally grown(?) fruits and vegetables than organic products that come from far away.
	Ground	Der CO2-Ausstoß, vor allem durch den nötigen Transport, ist in der Regel bei regionalen Produkten deutlich geringer als bei solchen, die von weit herkommen.	The CO2 emissions, especially resulting from essential transportation, are typically much lower for regional products compared to those that originate from distant locations.
	Warrant	Der Internationale Transfer von Waren wird überwiegend über den Schiff- und Flugverkehr realisiert, für die aktuell noch keine umweltfreundliche Treibstoffe Verwendung finden.	The international transportation of goods is predominantly carried out through ship and air transport, which currently do not utilize environmentally friendly fuels.
	Backing	Für den langen Transport müssen Bio-Obst und -Gemüse in deutlich mehr Plastik verpackt werden als regional konventionell Angebautes, was viel Müll produziert.	During long-distance transportation, organic fruits and vegetables require significantly more plastic packaging than conventionally grown regional produce, resulting in a substantial amount of waste.
	Rebuttal	Rein vom Anbau her betrachtet sind Bio-Produkte weniger schädlich für die Umwelt und damit nachhaltiger als konventionelle, unter anderem durch den Verzicht auf chemische Pestizide.	From an agricultural standpoint, organic products are less harmful to the environment and therefore more sustainable than conventional ones, primarily because they do not rely on chemical pesticides.
StS2	Claim	Maßnahmen gegen den Klimawandel belasten ökonomisch schwache Menschen finanziell überdurchschnittlich stark.	Measures to combat climate change place a unevenly high financial burden on economically weak people.

	Ground	Viele Maßnahmen zum Klimaschutz haben eine Verteuerung von Produkten zur Folge, da z.B. strengere Umweltauflagen oder zusätzliche Steuern die Produktionskosten erhöhen, die auf den Verbraucher abgewälzt werden.	Many climate protection measures lead to an increase in prices, since, for example, stricter environmental regulations or additional taxes increase production costs, which are passed on to the consumer.
	Warrant	Steigende Preise für alltäglich benötigte Produkte wirken sich bei geringem Einkommen stärker aus als bei hohem.	Rising prices for everyday products have a greater impact on people with low incomes than on people with high incomes.
	Backing	Ärmere Menschen können sich oft auch keine Alternative zu den sich verteuernden Produkten leisten. Sie müssen beispielsweise steigende Benzinpreise hinnehmen, da ein Elektroauto ohnehin unerschwinglich ist.	Poorer people often cannot afford alternative products, which are more expensive. For example, they have to get along with rising gasoline prices because an electric car is unaffordable to them.
	Rebuttal	Die finanzielle Belastung durch Klimaschutzmaßnahmen hängt stark von den Lebensgewohnheiten ab. Z.B. eine Benzinpreiserhöhung trifft Personen weniger, die den Arbeitsweg ohnehin mit dem Fahrrad zurücklegen.	The financial burden of climate protection measures depends heavily on one’s lifestyle. For instance, an increase in gasoline prices has a lesser impact on people who already commute to work by bicycle.
StS3	Claim	Eine Erweiterung der Möglichkeiten zur Forschung mit Gentechnik in Europa kann helfen, Nahrungssicherheit trotz des Klimawandels sicherzustellen.	Expanding the scope for research on genetic engineering in Europe can help to ensure food security despite climate change.
	Ground	Durch den Klimawandel werden sich aller Voraussicht nach Extremwetterereignisse häufen, und Nutzpflanzen müssen daran angepasst werden.	Climate change is likely to increase the frequency of extreme weather events, and crops will need to be adapted to resist them.
	Warrant	Würden die aktuell sehr strengen Regeln zum Umgang mit gentechnisch veränderten Pflanzen gelockert, könnte mehr Forschung betrieben werden und so mehr und bessere resistente Sorten entwickelt werden, um weiterhin genug Erträge zu erhalten und die Menschen ernähren zu können.	If the current highly stringent regulations on the use of genetically modified crops were eased, it could lead to more research opportunities to develop improved and more resistant varieties. This, in turn, could help maintain sufficient yields to feed the growing population.

	Backing	In einigen Ländern, in denen Nahrungsknappheit herrscht, tragen durch Gentechnik erzeugte angepasste Sorten schon heute klar zur Verbesserung der Situation bei.	In some countries with food shortages, genetically engineered adapted varieties have shown to improve the situation.
	Rebuttal	Wichtige Eigenschaften im Zusammenhang mit dem Klimawandel, vor allem Resistenz gegen Trockenheit, sind sehr schwer gentechnisch zu erzeugen und bis heute nicht erreicht.	Crucial traits related to climate change, particularly drought resistance, have been exceedingly challenging to genetically engineer and have not been successfully achieved up to the present time.
StS4	Claim	Es ist Aufgabe der Regierung, nicht das der einzelnen Bürger, nachhaltiges Verhalten zu veranlassen.	It is up to the government, not individual citizens, to promote sustainable lifestyles.
	Ground	Einzelne Personen haben oft keine Wahlmöglichkeit, da die umweltfreundlichere Option häufig deutlich teurer ist und die meisten Bürger bei komplexen Themen wie dem Klimawandel zu wenig Wissen haben, um ihr Handeln korrekt einzustufen.	Many individuals often find themselves without a choice, as the more environmentally friendly option is frequently considerably more expensive. Moreover, when dealing with complex issues like climate change, most citizens possess insufficient knowledge to accurately assess the impact of their actions.
	Warrant	Durch Verbote oder Verteuerung schädlicher Dinge, Förderung von umweltfreundlichen Alternativen und Aufklärungskampagnen kann die Politik die Sensibilität für das Thema Nachhaltigkeit bei Bürgern erhöhen und sie dazu motivieren, sich nachhaltiger zu verhalten.	Policymakers can play a vital role in raising awareness about sustainability issues among citizens and encouraging more sustainable behaviour. This can be achieved through measures such as banning or imposing higher costs on harmful items, promoting environmentally friendly alternatives, and conducting educational campaigns to inform the public about the importance of sustainability and the positive impact of their choices
	Backing	Maßnahmen zur Nachhaltigkeit, die die Industrie betreffen und die einen weit größeren Effekt auf die Nachhaltigkeit ausüben, als Maßnahmen im privaten Bereich, können nur vom Staat und nicht durch Privatpersonen veranlasst werden.	Sustainability measures that have a significant impact on sustainability and affect industries have to be initiated by the state rather than by private individuals.

	Rebuttal	Durch Maßnahmen wie Verbote o.ä. steht der Aspekt der Kontrolle stark im Vordergrund, was in einer demokratischen Gesellschaft wie Deutschland eher zu einer ablehnenden Haltung als zum erforderlichen gesellschaftlichen Umdenken führen kann.	Through measures such as bans or similar actions, the aspect of control is strongly emphasized, which in a democratic society like Germany can lead to a more rejecting attitude rather than the necessary societal shift in thinking.
StS5	Claim	Es wäre nicht nachhaltig, wenn alle Autofahrer sofort auf ein Elektrofahrzeug umsteigen würden.	It would not be sustainable if all car drivers switched to electric vehicles immediately.
	Ground	Aktuell gibt es zu wenig Strom aus erneuerbaren Energien, um den gesamten privaten Verkehrssektor damit zu betreiben.	Currently, there is too little electricity from renewable sources to fully power the entire private transport sector.
	Warrant	Wenn es nicht genug nachhaltigen Strom gibt, muss Strom aus fossilen Energieträgern genutzt werden, um den Bedarf zu decken, was dazu führt, dass Elektroautos nicht umweltfreundlicher sind als solche mit Verbrennungsmotor.	In the absence of sufficient sustainable electricity, the demand is met by using electricity generated from fossil fuels, rendering electric cars no more environmentally friendly than those with internal combustion engines.
	Backing	Berechnungen belegen, dass die Gewinnung von Strom aus erneuerbaren Quellen noch ca. 8 Jahre lang weiter ausgebaut werden muss, um den ganzen Privatverkehr nachhaltig auf Elektroautos umstellen zu können.	According to calculations, the expansion of electricity generation from renewable sources will need to continue for approximately 8 years before achieving a sustainable conversion of all private transport to electric cars.
	Rebuttal	Elektroautos mit Strom aus erneuerbaren Energien liegen in der Gesamtökobilanz weit vor Autos mit Verbrennungsmotor.	Electric cars powered by electricity from renewable sources have a significantly superior overall eco-balance compared to cars with combustion engines.
StE1	Claim	Traditionell wird in Deutschland das Stakeholder Konzept befürwortet, dagegen kommt in den USA eher das Shareholder Konzept zum Tragen.	Traditionally, the stakeholder concept is advocated in Germany, while the shareholder concept is more common in the USA.
	Ground	In Deutschland sind die meisten Unternehmen heute keine privaten Erwerbseinheiten eines oder mehrerer Eigentümer,	In Germany, most companies today are not private entities of one or more owners, but quasi-public institutions. They

		sondern quasiöffentliche Institutionen. Sie bilden einen Ort konfligierender (in Konflikt geratender) und komplementärer (ergänzender) Interessen unterschiedlicher unternehmensinterner und –externer Anspruchsgruppen und folgen demnach dem Stakeholder Konzept.	are a place of conflicting and complementary interests of various internal and external stakeholders and therefore follow the stakeholder concept.
	Warrant	Im deutschen System ist der Aufsichtsrat zur Hälfte von Seiten der Anteilseigner und zur anderen Hälfte von Arbeitnehmervertretern besetzt. In den USA steht eher der Anteilseigner im Vordergrund. Dadurch ist der Anteil der Arbeitnehmer im Aufsichtsrat wesentlich geringer als in Deutschland.	In the German system, half the members of the supervisory board are shareholder representatives and half are employee representatives. In the USA, the shareholder is more prominent. As a result, the proportion of employees on the board is much lower than in Germany.
	Backing	In den USA wird die Gewinnmaximierung deutlich höher als die soziale und gesellschaftliche Verantwortung des Unternehmens bewertet. Diese Differenz zeigt sich in Deutschland weniger stark und die soziale Verantwortung wird verstärkt wahrgenommen.	In the USA, profit maximization is rated much higher than the company's social responsibility. This difference is less pronounced in Germany, where social responsibility is more strongly perceived.
	Rebuttal	Durch die zunehmende Globalisierung entstehen vermehrt multinationale Unternehmen mit Standorten und Mitarbeitern aus verschiedensten Ländern. Die Ausrichtung und bevorzugten Konzepte der Unternehmen hängen daher nicht mit bestimmten Ländern zusammen. Beide Konzepte können je nach Kontext nutzbringend für die Gesellschaft sein.	Increasing globalization is leading to more multinational companies with operations and employees in many different countries. The orientation and preferred approaches of companies are therefore not tied to specific countries. Both approaches can be beneficial to society, depending on the context.
StE2	Claim	Der wirtschaftliche globale Aufschwung verliert an Kraft und führt zu einer gedämpften Wirtschaftsentwicklung in Deutschland.	The global economic upswing loses momentum, leading to subdued economic development in Germany.

	Ground	Die internationale Konjunktur hat sich zur Jahresmitte 2021 abgekühlt. Prognosestudien sagen eine Verlangsamung der weltwirtschaftlichen Produktion in den nächsten zwei Jahren voraus.	The international economy cooled off in mid-2021. Forecast studies predict a slowdown in global economic output over the next two years.
	Warrant	Die Verschlechterung des außenwirtschaftlichen Umfelds führt auch in Europa und vor allem Deutschland zu einer Wachstumsverlangsamung.	The deterioration in the external environment will also lead to a slowdown in growth in Europe and especially in Germany.
	Backing	Tragende Säule des Wachstums in Deutschland wird die Binnenwirtschaft sein, wobei die privaten Konsumausgaben die größte Antriebskraft sind. Die Ausweitung der Investitionstätigkeit bleibt trotz der anhaltend sehr günstigen Finanzierungsbedingungen sehr verhalten.	The mainstay of growth in Germany will be the domestic economy, with private consumption being the main driver. The expansion of investment activity will remain very subdued, despite continued very favorable financing conditions.
	Rebuttal	Der Welthandel dürfte im Prognosezeitraum von 2 Jahren weiter expandieren. Das BIP im Euroraum wird voraussichtlich in diesem Jahr um 1,5% und im nächsten um 1,6% zunehmen.	World trade is expected to continue to expand over the 2-year forecast period. GDP in the euro area is expected to grow by 1.5% this year and 1.6% next year.
StE3	Claim	Ein universeller moralischer Standpunkt ist bzw. universelle Standards sind für multinationale Unternehmungen in der Postmoderne unbegründbar.	A universal moral standpoint or universal standards are not tenable for multinational corporations in postmodernism.
	Ground	Der Vernunftskeptizismus des Postmodernismus beschreibt, dass universelle Begründungen im Gegensatz zur Moderne, in der rationale Entscheidungen durch universelle Begründungen (Wissenschaft) zentral waren, nicht mehr möglich sind.	The reason skepticism of postmodernism describes that universal justifications are no longer possible, in contrast to modernity, where rational decisions were central through universal justifications (science).

	Warrant	Aufgrund der Vielfalt an Rationalitäten und kulturellen Wertvorstellungen können keine umfassenden Begründungen und Standards mehr gesetzt werden. Die Tendenz geht daher zur Akzeptanz des Status quo.	Due to the diversity of rationalities and cultural values, universal justifications and standards are no longer possible. The tendency is therefore to accept the status quo.
	Backing	Der Nationalstaat als eigene Einheit verliert in der Postmoderne massiv an Bedeutung.	The nation-state as a separate entity loses massively in importance in postmodernity.
	Rebuttal	Für multinationale Unternehmen gibt es gute Gründe (z.B. die Steuerungsgrenzen der Gesetze, die Abhängigkeit von funktionierenden Märkten, die Verantwortungsübernahme als Teil des Risikomanagements) sich an der internationalen Regelsetzung zu beteiligen bzw. sich freiwillig zu Standards zu verpflichten.	For multinational companies, there are good reasons (e.g. the control limits of laws, the dependence on functioning markets, the assumption of responsibility as part of risk management) to participate in international rule-making or to voluntarily commit to standards.
StE4	Claim	Die Globalisierung bringt Effizienzvorteile für alle Länder.	Die Globalisierung bringt Effizienzgewinne für alle Länder.
	Ground	Die globale Allokation (Zuweisung von finanziellen Mitteln, Materialien und Produktivkräften) der vorhandenen Ressourcen ist effizienter als die lokale.	Die globale Allokation (Zuweisung von Finanzmitteln, Materialien und Produktivkräften) der verfügbaren Ressourcen ist effizienter als die lokale Allokation.
	Warrant	Ein besseres Ressourcenmanagement ermöglicht den Entwicklungs- und Schwellenländern ihre Kostenvorteile zur Geltung zu bringen.	Ein besseres Ressourcenmanagement ermöglicht es den Entwicklungs- und Schwellenländern, ihre Kostenvorteile zu nutzen.
	Backing	Die Anpassung politischer Rahmenbedingungen an die Erfordernisse der Wirtschaft verringert die Ineffizienzen von politischen Systemen.	Die Ausrichtung des politischen Rahmens an den Bedürfnissen der Wirtschaft verringert die Ineffizienz der politischen Systeme.
	Rebuttal	Eine Erhöhung von Arbeits-, Sozial- und Umweltstandards mindert die Kostenvorteile der Entwicklungsländer.	Die Anhebung von Arbeits-, Sozial- und Umweltstandards verringert den Kostenvorteil der Entwicklungsländer.

StE5	Claim	Die Globalisierung der Wirtschaft bringt neben Vorteilen auch Nachteile für viele Bürger.	Economic globalization brings both advantages and disadvantages to many citizens.
	Ground	Die Effizienzgewinne werden ungleich verteilt und die Globalisierung stellt eine Gefahr für die Funktionsfähigkeit und die Legitimation des politischen Systems dar.	Efficiency gains are unevenly distributed, and globalization poses a threat to the functioning and legitimacy of the political system.
	Warrant	Die Globalisierung braucht einen politischen Rahmen, der sich auf eine ökonomieunabhängige Begründung stützt, um eine bessere Verteilung von Effizienzgewinnen zu erreichen.	Globalization needs a political framework based on non-economic principles to achieve a better distribution of efficiency gains.
	Backing	Die sich vertiefende internationale Arbeitsteilung führt zu zwei Problemkreisen: mangelnde „Fairness“ durch künstliche Wettbewerbsvorteile (verursacht durch Manipulation der Wirtschaftspolitik) und die fortschreitende wirtschaftliche Integration, die zu einer Verarmung der unqualifizierten Arbeitskräfte in den reichen Ländern führt.	The deepening international division of labor leads to two sets of problems: lack of "fairness" due to artificial competitive advantages (caused by the manipulation of economic policies), and progressive economic integration leading to the impoverishment of unskilled labor in rich countries.
	Rebuttal	Eine bessere Zuweisung von finanziellen Mitteln, Materialien und Produktivkräften durch die Globalisierung ermöglicht den Entwicklungs- und Schwellenländern, ihre Kostenvorteile zur Geltung zu bringen.	Better allocation of financial resources, materials and productive forces through globalization allows developing and emerging countries to exploit their cost advantages.

Supplementary Material 2. Items for the recognition of fallacies

Item	Fallacy	German (original)	English (translated)
FaP1	No fallacy	Konstruktivistisch orientierte Lernsettings eignen sich besonders gut, um den Prozess der Umstrukturierung von naiven kindlichen Alltagsvorstellungen beim naturwissenschaftsbezogenen Lernen zu fördern. Gemäß der Conceptual-Change-Forschung kann eine Weiterentwicklung von Konzepten dann stattfinden, wenn die Lernenden durch aktiven Umgang mit Phänomenen die Grenzen ihrer Vorstellungen erkennen und zu einer Deutung gelangen, die zu ihren Beobachtungen passt. Ein konstruktivistisch orientierter Unterricht schafft Lerngelegenheiten, in denen die Lernenden eigene Vermutungen aufstellen, Erprobungen durchführen, Beobachtungen anstellen und Schlüsse ziehen können. Dadurch können sie ihre Vorstellungen entwickeln, überprüfen und verändern.	Constructivist-oriented learning environments are highly effective in facilitating the transformation of children's naive everyday conceptions during science learning. Conceptual change research suggests that learners can develop new understandings when they actively engage with phenomena, recognize the limitations of their existing conceptions, and arrive at interpretations that align with their observations. In a constructivist-oriented classroom, students are provided with learning opportunities that encourage them to form their own hypotheses, conduct experiments, make observations, and draw conclusions. This process enables them to continually develop, test, and refine their ideas.
FaP2	No fallacy	Studierende haben oft Schwierigkeiten, Fachtexte zu erschließen, relevante Aspekte zu identifizieren und diese adäquat in eigene Worte zu fassen. Deshalb werden Textzusammenfassungen als gängige Schreibaufgabe für Studierende unterer Semester eingesetzt. Diese Aufgabe erfordert es, die eigene Wortwahl und Satzkonstruktion auf den Prüfstand zu stellen, was viel Zeit in Anspruch nimmt. Studienergebnisse zeigen jedoch, dass diese Fähigkeiten durch regelmäßiges Üben verbessert werden können und die intensive Auseinandersetzung mit dem Textgegenstand und	Students often face difficulties when it comes to comprehending specialized texts, extracting relevant information, and effectively expressing it in their own words. To address this, text summaries are frequently assigned as writing tasks for lower-level students. This exercise not only helps students refine their word selection and sentence structure but also demands considerable time and effort. Nonetheless, research demonstrates that regular practice can lead to significant improvement in these skills. Moreover, delving deeply into the text's content and one's writing abilities fosters a more comprehensive understanding of the content.

		der eigenen Schreibkompetenz ein detailliertes Verständnis für den Textinhalt fördert.	
FaP3	Circularity	Wichtigstes Ziel eines Lernprozesses ist es, dass Lernende kompetentes Verhalten zunehmend von sich aus zeigen und am Ende möglichst ganz ohne Unterstützung auskommen. Während neue Arbeitsschritte für den Lernenden zunächst nur mit Hilfe des Lehrenden durchgeführt werden können, werden mit zunehmendem Kompetenzniveau weniger Hilfestellungen benötigt. Das Nachlassen von Unterstützung durch den Lehrenden wird als Fading bezeichnet. Im Prozess der Aufgabenübernahme zieht sich der Lehrende demnach zunehmend zurück, bis der Lernende in der Lage ist, die Lösungen selbständig zu erarbeiten. Beim Fading handelt es sich also um einen Prozess, bei dem im Verlauf des Lernvorganges lernunterstützende Maßnahmen stufenweise abgebaut werden.	The primary objective of a learning process is to enable the learner to develop increasing competence and eventually perform tasks independently, without any assistance. In the initial stages, learners may require help from the teacher to grasp new concepts and skills. However, as their competence grows, the need for assistance diminishes. This gradual reduction in teacher support is referred to as "fading". During this process, the teacher gradually withdraws his or her involvement, allowing the learner to work out solutions on their own. Essentially, fading involves a gradual decrease in learning support as the learner progresses through the learning journey
FaP4	Overgeneralization	Das Lernen am Modell zeichnet sich zum einen durch die bewusste Beobachtung durch den Lernenden und zum anderen durch eine kognitive Verarbeitung der beobachteten Reize aus. Dabei muss die Aufmerksamkeit des Lernenden auf das Modell gelenkt sein, um das Interesse an der zu beobachtenden Situation zu wecken. Diese Annahme wird durch die sehr häufig zitierte Bobo-Doll-Studie bewiesen, in der Kinder spezifische Verhaltensweisen nur dann zeigten, wenn sie diese zuvor bei erwachsenen Vorbildern beobachtet haben. Das Verhalten wurde demnach durch die Beobachtung des Modells und die damit verbundene kognitive Verarbeitung des Gesehenen erlernt.	Social learning theory emphasizes the importance of conscious observation and cognitive processing of stimuli by the learner. To facilitate this process, the learner's attention must be directed towards the social model to generate interest in the observed situation. The well-known Bobo Doll study provides evidence for this assumption, as children displayed specific behaviors only after witnessing them in adult models, indicating that learning occurred through observation and cognitive processing of the observed actions. Applying this theory to an educational context, when a teacher serves as the model and demonstrates task steps on the blackboard, it is crucial for the student's attention to

		Wenn demnach eine Lehrperson (als Modell) Aufgabenschritte an der Tafel vormacht, muss die Aufmerksamkeit des Schülers auf die Lehrperson gerichtet sein. Nur so wird eine kognitive Verarbeitung gewährleistet und der Schüler dazu befähigt, die Lösungsschritte im Anschluss eigenständig durchzuführen.	be focused on the teacher. This ensures that the cognitive processing takes place effectively, enabling the student to perform the solution steps independently later on.
FaP5	Formal fallacy	Wie Studien aus der Motivationsforschung zeigen, ist die Leistungsmotivation bei Lernenden mit hoher Zuversicht für Erfolg bei schwierigeren Aufgaben am höchsten. Eine Erklärung dafür liefert das Erwartungs-Wert-Modell: Die Motivation ist dann am höchsten, wenn der Lernende sowohl eine hohe Erwartung auf Erfolg hat sowie dem Lösen der Aufgabe einen hohen Wert zumisst. Je schwieriger die Aufgabe, desto eher verringert sich die Erwartung, die Aufgabe lösen zu können. Gleichzeitig erhöht sich der Wert des Erfolges, weil man auf den Erfolg stolz sein kann. In Experimenten, in denen Lernende zwischen Aufgaben unterschiedlicher Schwierigkeit wählen durften, wählten Personen mit hoher Zuversicht auf Erfolg signifikant häufiger schwierige Aufgaben. Diese Ergebnisse lassen darauf schließen, dass Lernende mit einer niedrigen Erfolgszuversicht eher leichtere Aufgaben wählen, da dort die Motivation für sie am höchsten ist.	Studies in motivation research have revealed that learners with a strong belief in their ability to succeed (high expectancy of success) exhibit the highest achievement motivation when confronted with more challenging tasks. This phenomenon can be explained by the expectancy-value model, which suggests that motivation is at its peak when the learner possesses both a high expectancy of success and a high value for completing the task. As tasks become more difficult, the expectation of success may decrease; however, simultaneously, the value attached to achieving success increases due to the sense of accomplishment and pride associated with overcoming challenges. In experiments where learners were given a choice between tasks of varying difficulty levels, individuals with high confidence in their ability to succeed were significantly more inclined to opt for difficult tasks. Consequently, it can be inferred that learners with lower confidence in their ability to succeed are more likely to choose easier tasks, as their motivation is highest in such scenarios.
FaP6	Irrelevance	Dass Kinder lernen, logisch und problemlösungsorientiert zu denken, ist ein pädagogisches Ziel, welches im Zeitalter des immer schneller werdenden digitalen Wandels eine	The development of logical thinking and problem-solving skills in children has become increasingly crucial in our rapidly evolving digital era. Chancellor Merkel has emphasized the importance

		zunehmend größere Bedeutung erhält. Bundeskanzlerin Merkel bezeichnet die Fähigkeit "Coden" zu können als Basisfähigkeit, die Schülern nebst Lesen und Schreiben an der Schule lernen sollten. Zur Erreichung dieses Ziels muss die flächendeckende digitale Ausstattung von Schulen weiter vorangetrieben werden. Beispielsweise durch den Einsatz des pädagogischen Konzepts des Computational Thinking kann das problem- und lösungsorientierte Denken bereits bei Grundschülern nachweislich gefördert werden. Die Einbettung dieser Kompetenzen in die bestehenden Lehrpläne ist daher langfristig unabdingbar. Dies zeigt sich bereits in den Vorreiterländern wie z.B. England oder Australien, in denen Computational Thinking bereits ab der ersten Klasse in die Curricula verankert wurde.	of teaching students the ability to "code," considering it a fundamental skill on par with reading and writing. To achieve this educational objective, schools must advance their digital infrastructure comprehensively. The implementation of computational thinking as a pedagogical concept has shown to effectively promote problem-solving and solution-oriented thinking, even among elementary school students. Therefore, it is essential to integrate these competencies into existing curricula for long-term success. Pioneering countries like England and Australia have already recognized this significance and have successfully embedded computational thinking into their curricula from the first grade onward.
FaS1	No fallacy	Viele Fluggesellschaften bieten bei der Buchung von Flügen die Möglichkeit, einen kleinen Teil der dabei entstehenden CO2-Emissionen durch eine Zahlung von wenigen Euro auszugleichen, sogenannte Klimakompensationen. Analysen von Passagierzahlen und anschließende Befragungen haben ergeben, dass durch diese Möglichkeit die Kunden häufiger fliegen als zuvor und so insgesamt mehr CO2 produzieren. Dies lässt sich damit erklären, dass sie ein gutes Gewissen bekommen und annehmen, Flugreisen nicht mehr vermeiden zu müssen aus Sicht des Klimaschutzes. Deshalb haben diese Zahlungen insgesamt gesehen einen negativen Effekt auf unser Umweltschutzverhalten und sollten verboten statt ihr Angebot ausgeweitet werden, wenn man tatsächlich etwas für das Klima tun will.	When booking a flight, several airlines provide the choice of paying a small fee to offset a portion of the resulting CO2 emissions. However, studies based on passenger numbers and subsequent surveys have indicated that this option actually leads customers to fly more frequently than before, resulting in an overall increase in CO2 emissions. The rationale behind this behavior lies in the belief that by making the offset payment, they have fulfilled their climate protection responsibility and no longer need to avoid air travel. Therefore, if we genuinely want to make a positive impact on the climate, they should be banned instead of expanded.

FaS2	No fallacy	Die Nutzung von Baumwolltragetaschen als Alternative zu Plastiktüten ist ökologisch gesehen weniger effektiv, als viele Menschen glauben. Unter dem Aspekt der Entsorgung belasten Baumwolltaschen zwar aufgrund ihrer deutlich niedrigeren Verrottungsdauer die Umwelt weniger als Plastiktüten. Aufgrund des ressourcenintensiven Anbaus und der aufwändigen Herstellung schneiden Baumwolltaschen nach der Gesamtbilanz des Bundesumweltamtes jedoch deutlich schlechter ab als Plastiktüten, die vorwiegend aus Recyclingmaterial hergestellt werden. Erst ab einer 83-maligen Wiederverwendung sinkt die relative Belastung der Umwelt durch eine Baumwolltasche auf ein Niveau, das mit dem einer Plastiktüte nach einmaligem Gebrauch vergleichbar ist. Hochrechnungen der Gesellschaft für Verpackungsforschung zufolge liegt die durchschnittliche Wiederverwendungsrate allerdings weit unter dieser Marke.	Using cotton bags as a replacement for plastic bags might not be as environmentally friendly as many people believe. From a disposal standpoint, cotton bags do indeed have a lower environmental impact because they decompose faster than plastic bags. However, their cultivation and production process are resource-intensive and costly, making them less environmentally friendly compared to plastic bags, which can often be made from recycled materials, as per the German Federal Environment Agency's Life Cycle Assessment. To equal the environmental impact of a single-use plastic bag, a cotton bag needs to be reused 83 times. However, according to the German Society for Packaging Research, the average reuse rate of cotton bags falls well below this mark.
FaS3	Circularity	Berechnungen des Bundesumweltamtes zufolge sind mehr als 65% der klimaschädlichen CO2-Emissionen in Deutschland auf den Verkehrssektor zurückzuführen. Eine Verringerung des CO2-Ausstoßes durch Autos würde zwar CO2-Emissionen nicht vollständig eindämmen, uns jedoch dem Klimaziel ein großes Stück näherbringen. Vor diesem Hintergrund sollten möglichst viele Menschen bald auf umweltfreundlichere Alternativen zum Verbrennungsmotor umsteigen, die kein CO2 ausstoßen. Beispielsweise Elektroautos bilden beim Fahren keine klimaschädlichen Emissionen, weshalb	According to the German Federal Environment Agency, over 65% of climate-damaging CO2 emissions in Germany originate from the transportation sector. While reducing CO2 emissions from cars alone might not completely solve the issue, it would significantly contribute to achieving climate goals. To move closer to these objectives, a widespread shift to more environmentally friendly alternatives to internal combustion engines, which produce no CO2 emissions, is essential. Electric cars, for instance, emit no climate-damaging emissions during operation, making them a promising solution for the

		diese zukünftig mehr genutzt werden sollten. Je mehr Menschen daher beim nächsten Autokauf auf emissionsfreie Autos setzen, desto weniger wird die Umwelt durch den CO2-Ausstoß von Verbrennungsmotoren belastet.	future. Encouraging as many people as possible to choose zero-emission cars when purchasing their next vehicle would substantially decrease the environmental impact of CO2 emissions from internal combustion engines.
FaS4	Overgeneralization	Eine Studie mit Studierenden der Energietechnik zeigt, dass Studierende, die ein Zusatzseminar über die Begrenztheit der Ressource Wasser und die Bedeutung des Wassersparens im Alltag besuchten, sich anschließend auch in anderen Lebensbereichen nachhaltiger verhalten haben. Die Einbindung eines Seminares in alle Studiengänge, in dem über einen beliebigen Aspekt der Nachhaltigkeit informiert wird, würde Studierende demnach zu einem allgemein umweltbewussteren Verhalten bewegen. Zwar gab es auch Fälle, in denen die im Seminar vermittelten Inhalte nicht beachtet wurden oder sich sogar gegenteilig verhalten wurde. Die Mehrheit der Studierenden wendete das Gelernte allerdings sinnvoll an und konnte dies sogar erfolgreich auf andere Bereiche übertragen.	The study of energy engineering students revealed that those who participated in an additional seminar focusing on the finite nature of water as a resource and the importance of water conservation in daily life displayed more sustainable behaviour in various aspects of their lives. This finding suggests that incorporating sustainability seminars into all undergraduate programs could foster a broader environmental consciousness among students. While it is true that there were instances where the content of the seminar was not followed, and even opposite behaviours were observed, the majority of students effectively applied what they had learned and successfully transferred their knowledge to other areas of their lives. This indicates that the impact of such seminars goes beyond the specific topic taught and can lead to meaningful and positive changes in overall behaviour.
FaS5	Formal fallacy	Eine Untersuchung im Auftrag der Bundesregierung ergab, dass Menschen ein höheres Naturbewusstsein entwickeln, wenn sie täglich zehn Minuten entspannende Naturvideos ansehen. Dies ist dadurch begründet, dass die regelmäßige Wiederholung der Darbietung und der positive Kontext der Entspannung eine verbesserte und positivere Einstellung gegenüber dem Gesehenen erzeugen. Diese Ergebnisse lassen darauf schließen, dass Menschen, die regelmäßig ruhige Videos von	A study commissioned by the German government revealed that watching relaxing nature videos for just ten minutes a day leads to a greater awareness and appreciation of nature. This effect is attributed to the regular exposure and positive context of relaxation that fosters a more positive attitude towards the natural scenes being viewed. Conversely, the study suggests that people who regularly watch calming videos of urban environments may develop a reduced awareness of

		urbanen Umgebungen ansehen, ein verringertes Naturbewusstsein aufweisen. Sie fühlen dann eine stärkere Verbundenheit mit dem im Video dargebotenen Raum und grenzen sich tendenziell von Gegenteiligem ab. Dieser Effekt tritt demnach nicht ein, wenn bei der Darbietung urbaner Videos ausreichend natürliche Elemente wie Grünflächen enthalten sind.	nature. This is because they become more attached to the urban space presented in the videos and tend to disconnect themselves from anything contrary to that environment. However, the study also found that this effect is mitigated when sufficient natural elements, such as green spaces, are included in the presentation of urban videos.
FaS6	Irrelevance	Das vom Bundestag verabschiedete Plastiktüten-Verbot, welches ab 2022 in Kraft tritt, ist ein bedeutender Schritt auf dem Weg zu einer nachhaltigen Gesellschaft. Bereits die Initiative von Lebensmittelmärkten zur Abschaffung von Plastiktüten wurde von Umweltministerin Hendricks sehr befürwortet und als wichtiges Signal an die Gesellschaft gelobt. Es ist zwar unstrittig, dass das Plastiktüten-Verbot allein nicht ausreicht, um alle derzeitigen Umweltprobleme zu lösen. Da das Einkaufen allerdings das tagtägliche Leben aller Menschen betrifft, kann diesem Verbot aufgrund seiner sich summierenden Wirkung eine besondere Bedeutung beigemessen werden. Wenn man sich allein vorstellt, dass in Deutschland rund 80 Millionen Menschen bei ihrem täglichen Einkauf mindestens eine Plastiktüte verwenden, wird ein Verbot maßgeblich zur Verbesserung der Umwelt beitragen.	The plastic bag ban approved by the German Bundestag, set to be enforced in 2022, represents a significant stride towards building a more sustainable society. Grocery stores' initiative to phase out plastic bags has garnered substantial support, including praise from Environment Minister Hendricks, who sees it as a crucial signal to society. While it is true that banning plastic bags alone cannot address all of today's environmental challenges, its impact is notable due to the widespread use of plastic bags during shopping, affecting everyone's daily life. With approximately 80 million people in Germany utilizing at least one plastic bag for their daily shopping, the ban will make a considerable contribution to environmental improvement.
FaE1	No fallacy	Kommendes Jahr werden wir mehr intelligente Lautsprecher, mehr gesprächsbereite künstliche Intelligenz und damit auch mehr Anreize zu neuen Kommunikationsideen erleben. Es ist das Jahr, in dem Händler wie Tink herausfinden wollen, wie bereit die Durchschnittsverbraucher tatsächlich für	Next year, we'll see more smart speakers, more conversational artificial intelligence, and more incentives for new communication ideas. It's the year that retailers like Tink want to find out just how ready the average consumer really is for the smart home. After all, if people are going to continue to

		das Smart Home sind. Denn damit die Menschen auch nach dem ersten Hypecycle das Gespräch mit Alexa, Google Agent und Siri suchen, müssen diese mehr können, als Musik zu spielen, Suchmaschinenanfragen auszuführen und gelegentlich mal einen Witz zu erzählen. Soll das Internet der Dinge tatsächlich ein nachhaltiger Trend und damit ein spannendes neues Wachstumsfeld für das Marketing werden, müssen seine Produkte durch Nützlichkeit überzeugen.	seek out conversations with Alexa, Google Agent, and Siri after the initial hypercycle, they'll need to be able to do more than play music, perform search engine queries, and tell the occasional joke. If the Internet of Things is truly going to become a sustainable trend, and thus an exciting new growth area for marketing, its products will need to deliver utility.
FaE2	No fallacy	Content Marketing ist seit vielen Jahren ein effektives Kommunikationsmittel für Unternehmen. Laut Studie führen in Deutschland 69% der befragten Unternehmen eine gut entwickelte und klare Content-Marketing-Strategie. 68% geben an, ihre Content Marketing Strategien auch langfristig zu planen. Erfolgreiches Content Marketing hängt unter anderem von mehreren Faktoren ab: Klarheit und Commitment bezüglich der Content Marketing Strategie, eine auf die Bedürfnisse der Zielgruppe abgestimmte Content-Produktion sowie normative journalistische Qualitätskriterien. 26% der befragten Unternehmen geben jedoch an, dass sie den Schwerpunkt auf alternative Strategien wie der Entwicklung einer Buyer Persona, der Optimierung der sprachbasierten Suchtechnologie oder einer Native Advertising Campaign legen, um sich so von Konkurrenten abzuheben.	Content marketing has been an effective communication tool for businesses for many years. According to a study, 69% of companies surveyed in Germany have a well-developed and clear content marketing strategy. 68% also say they plan their content marketing strategies for the long term. Successful content marketing depends on several factors, including Clarity and commitment to the content marketing strategy, content production that is tailored to the needs of the target audience, and normative journalistic quality criteria. However, 26% of companies surveyed say they are focusing on alternative strategies such as developing a buyer persona, optimizing voice-based search technology, or running a native advertising campaign to differentiate themselves from competitors.
FaE3	Circularity	Die aktuellen Entwicklungen zeigen, dass Controller zukünftig zunehmend eine beratende Funktion als Businesspartner bei komplexen betriebswirtschaftlichen und produktbezogenen Fragestellungen für das Management einnehmen, da	Current trends indicate that controllers will increasingly take on a consulting role as business partners to management on complex business and product-related issues. They will become initiators and facilitators of change and learning processes,

		sie in den meisten Unternehmen in den letzten Jahren vorrangig als Berater des Managements für Prozessabläufe agiert haben. Sie werden als Initiatoren und Moderatoren von Veränderungs- und Lernprozessen agieren, als Architekten von Informationslandschaften fungieren sowie zu Spezialisten für Prozessabläufe werden. Allerdings werden in Konkurrenz zu diesen Entwicklungen im Aufgabenprofil des Controllers auch neue oder weiterentwickelte Instrumente und Methoden an Bedeutung gewinnen.	architects of information landscapes, and specialists in process flows. In competition with these developments in the controller's job profile, new or improved tools and methods will also gain in importance.
FaE4	Overgeneralization	Eine aktuelle Studie von 2021 hat die Digitalisierung der Arbeitswelt in Deutschland untersucht und einen inzwischen großen Nachholbedarf kleiner und mittlerer Unternehmen (KMU) bei der digitalen Arbeit offenbart. Es wird deutlich, dass über Jahre hinweg die betriebliche digitale Transformation weltweit fatalerweise nur mühsam voranschritt. Dabei geht es um die Arbeit, die die allermeisten von uns jeden Tag ganz persönlich betrifft. Denn nicht erst seit der Corona-Pandemie und den damit verbundenen Herausforderungen für viele Betriebe gilt: Digitale Plattformen sowie das Internet der Dinge mit seinen intelligenten und vernetzten Produkten werden die digitale Transformation von Wirtschaft und Gesellschaft vorantreiben. Die Corona-Pandemie hat allerdings in den letzten 1,5 Jahren auch zu einem massiven Vorankommen der Digitalisierung in kleinen und mittleren Unternehmen geführt, da ein produktives Arbeiten ansonsten kaum noch möglich gewesen wäre.	A recent study by 2021 examined the digitization of the workplace in Germany and found that small and medium-sized enterprises (SMEs) have a lot of catching up to do when it comes to digital work. It shows that the digital transformation of companies worldwide has been painfully slow for years. We are talking about work that affects the vast majority of us personally every day.<br>Not only since the Corona pandemic and the associated challenges for many companies the following has been true: Digital platforms and the Internet of Things with its intelligent and connected products will drive the digital transformation of the economy and society. However, the Corona pandemic has also led to a massive advance in digitization in small and medium-sized enterprises over the past 1.5 years, as productive work would otherwise have been virtually impossible.

FaE5	Formal fallacy	Das Thema Mitarbeiterbindung hat sich zu einer der zentralen Herausforderungen im demografischen Wandel entwickelt und Feelgood-Management gehört inzwischen auch zum guten HR-Repertoire. Die herkömmlichen Argumente „Gehalt und Urlaubstage“ wirken laut einer Umfrage unter Mitarbeitern verschiedener Unternehmen kaum noch überzeugend. Daher scheint vor allem die Vereinbarkeit von Familie und Beruf sowie die in Bezug auf Ort und Zeit flexible Arbeitsgestaltung ausschlaggebend zu sein. Der Personaler fungiert also in weiten Teilen als Feelgood-Manager. Aber noch nicht alle Unternehmen basieren auf modernen Personalstrukturen und vor allen in Unternehmen mit einem höheren Durchschnittsalter spielen herkömmliche Argumente noch eine bedeutende Rolle.	Employee retention has become one of the key challenges of demographic change, and feel-good management is now part of the repertoire of good HR practice. According to a survey of employees at various companies, the traditional arguments of "salary and vacation days" no longer seem convincing. What seems to matter more is the ability to balance family and career, as well as the flexibility of working hours and location. To a large extent, the HR manager therefore acts as a feel-good manager. But not all companies are based on modern HR structures, and traditional arguments still play an important role, especially in companies with a higher average age.
FaE6	Irrelevance	Es ist als Führungskraft wichtig, sachlich klar und wertschätzend zu kommunizieren, um die Mitarbeiter zu erreichen, persönlich, per Telefon und Internet. Die Komplexität in der Art der Kommunikation der heutigen Zeit steigt, weil sich die Rahmenbedingungen verändern. Das macht Kommunikation als Führungsinstrument noch wichtiger. Einer Studie in Deutschland kam zu dem Ergebnis, dass Führung ganz unterschiedlich wahrgenommen wird. Während 62 % der befragten Führungskräfte überzeugt waren, ihre Mitarbeiter zu inspirieren und zu fördern, waren diese nur zu 36 % davon überzeugt. Nichtsdestotrotz gaben 76% der Mitarbeiter an, grundsätzlich zufrieden mit dem Führungsstil ihrer Vorgesetzten zu sein.	As a manager, it is important to communicate in a factual, clear, and appreciative manner to reach employees in person, on the phone, and on the Internet. The complexity of communication in today's world is increasing as the environment changes. This makes communication even more important as a management tool. A study in Germany found that perceptions of leadership vary widely. While 62% of managers surveyed thought they inspired and encouraged their employees, only 36% of them thought they did. Despite this, 76% of employees said they were generally satisfied with their manager's leadership style.
